# Identification of drought stress-responsive transcription factors in ramie (*Boehmeria nivea L. Gaud*)

**DOI:** 10.1186/1471-2229-13-130

**Published:** 2013-09-10

**Authors:** Touming Liu, Siyuan Zhu, Qingming Tang, Yongting Yu, Shouwei Tang

**Affiliations:** 1 Institute of Bast Fiber Crops and Center of Southern Economic Crops, Chinese Academy of Agricultural Sciences, Changsha 410205, China

**Keywords:** Ramie, Drought stress, Transcription factor, Illumina tag-sequencing, qRT-PCR

## Abstract

**Background:**

Ramie fiber extracted from stem bark is one of the most important natural fibers. Drought is a main environment stress which severely inhibits the stem growth of ramie and leads to a decrease of the fiber yield. The drought stress-regulatory mechanism of ramie is poorly understood.

**Result:**

Using Illumina sequencing, approximately 4.8 and 4.7 million (M) 21-nt cDNA tags were respectively sequenced in the cDNA libraries derived from the drought-stressed ramie (DS) and the control ramie under well water condition (CO). The tags generated from the two libraries were aligned with ramie transcriptome to annotate their function and a total of 23,912 and 22,826 ramie genes were matched by these tags of DS and CO library, respectively. Comparison of gene expression level between CO and DS ramie based on the differences of tag frequencies appearing in the two libraries revealed that there were 1516 potential drought stress-responsive genes, in which 24 genes function as transcription factor (TF). Among these 24 TFs, the unigene19721 encoding the DELLA protein which is a key negative regulator in gibberellins (GAs) signal pathway was probably markedly up-regulated under water stress for a increase of tag abundance in DS library, which is possibly responsible for the inhibition of the growth of drought-stressed ramie. In order to validate the change of expression of these potential stress-responsive TFs under water deficit condition, the unigene19721 and another eleven potential stress-responsive TFs were chosen for further expression analysis in well-watered and drought-stressed ramie by real-time quantitative PCR (qRT-PCR) and the result showed that all 12 TFs were authentically involved in the response of drought stress.

**Conclusion:**

In this study, twelve TFs involving in the response of drought stress were first found by Illumina tag-sequencing and qRT-PCR in ramie. The discovery of these drought stress-responsive TFs will be helpful for further understanding the drought stress-regulatory mechanism of ramie and improving the drought tolerance ability of ramie.

## Background

Drought is one of the most common environmental stresses that affect the growth and development of plants [1]. The global scarcity of water resources has already become a severe environmental problem worldwide. Poor water management, increased competition for limited water resources, and the uncertain threats associated with global warming all highlight the looming water crisis that threatens agricultural productivity worldwide. It has become urgent to elucidate the responses and adaptation of crops to water stress, and improve the drought tolerance of crops.

Plant response to drought stress is a complex course, and several mechanisms known as drought escape, drought avoidance and drought tolerance are involved in adapting the environment of water deficit [2]. A great number of dynamic responses to water deficit at physiological, biochemical, and molecular levels are presented in plant, thus enabling them to survive under drought environmental conditions [3,4]. Recently, expanding transcriptome data sets have uncovered a global picture of stress responsive genes in Arabidopsis [5], rice [6], maize [7], wheat [8] and other plants. These transcriptome data revealed that drought stress induced genes not only function to protect cells from drought stress through the production of important enzymes and metabolic proteins (functional proteins), but they also regulate signal transduction and gene expression in the stress response (regulatory proteins). The functional proteins include late embryogenesis abundant (LEA) proteins, a variety of transporters, enzymes involved in osmoprotectant synthesis, fatty acid metabolism, cellular metabolism, carbohydrate metabolism and secondary metabolism. Regulatory proteins that are activated in response to water stress, including transcription factors (TFs) such as DREBs (dehydration-responsive element-binding proteins), AREBs (ABA-responsive element-binding proteins) and NAC proteins, have been identified in plant [4,9,10]. Besides, many genes involved in growth and development, such as chloroplast, cell wall and plasma membrane proteins encoded gene, were down-regulated in response to drought stress [10].

Ramie (*Boehmeria nivea*), popularly called “China grass”, is one of the most important natural fiber crops. Ramie fibers, which are extracted from stem bast, are smooth, long and have excellent tensile strength. This high fiber quality is the major reason that ramie is widely cultivated in China, India, and other Southeast Asian and Pacific Rim countries. In China, ramie is the second most important fiber crop, with its growth acreage and fiber production being second only to those of cotton. Ramie has vigorous vegetative growth and can be harvested three times per year in China, and up to six times per year in well-watered cultivation environments in Philippines, which makes ramie produce a high yield of vegetative fiber. Therefore, enough water supplied by growing environment is essential to meet the requirement of vigorous metabolism for vegetative growth. When ramie suffered from water deficit, there were numbers of morphological and physiological changes in response to drought stress, such as leaf and root shape, malondialdehyde and proline contents, catalase activity and net photosynthetic rate in ramie [11]. However, up to now, none of genes involved in drought tolerance was identified and the potential drought stress-regulatory mechanism is still unknown in ramie. In this study, in order to identify the drought stress-responsive transcription regulator, the potential stress-responsive genes were identified on the basis of Illumina tag-sequencing at first; and then the differentially expressed TFs were screened and further validated by qRT-PCR. This study will be helpful for further elucidating the potential molecular responsive mechanism of ramie to drought stress and improving the drought tolerance ability of ramie.

## Result

### Stem traits and fiber yield of ramie in response to drought stress

**Figure 1:**
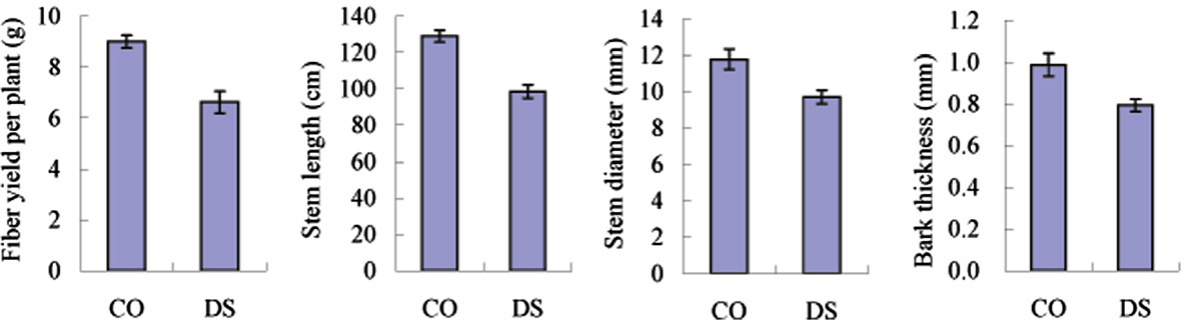
**The changes of ramie fiber yield and stem traits in response to drought stress.** The error bar represented the standard error.

Under well water condition, the stem length, diameter and bark thickness were 128.9 cm, 11.79 mm and 0.987 mm, respectively (Figure 1); whereas significant decreases in stem length, diameter and bark thickness (98.6 cm, 9.70 mm and 0.793 mm, respectively) were observed when ramie suffered from drought stress (Figure 1). Besides, the fiber yield of drought-stressed ramie (6.62 g per plant) was far lower than that of well-watered ramie (8.99 g per plant) (Figure 1). Therefore, drought environment severely inhibits the stem growth of ramie and leads to a decrease of the fiber yield.

### Tag identification and quantification

A total of 4,719,982 and 4,804,046 tags were sequenced in control ramie (CO) and drought-stressed ramie (DS) libraries, respectively (Table 1). After filtering out low quality tags (tags containing ‘N’ and adaptor sequences), 4,715,625 and 4,799,759 tags (noted herein as “clean” tags) remained in CO and DS libraries. To increase the robustness of the approach, single-copy tags in the two libraries (305,492 in CO and 319,431 in DS library) were excluded from further analysis. As a result, a total of 4,410,133 (93.44%) and 4,480,328 (93.26%) clean tags remained in the two libraries, from which 328,806 (CO) and 340,187 (DS) unique tags were obtained. Hence, only 6.56% and 6.74% tags in CO and DS libraries respectively were useless, which suggested that the sequence quality was excellent in the two libraries. There were 11,381 more unique tags in DS library than in the CO library, possibly representing genes related to drought tolerance.

**Table 1:** Illumina tags in the control (CO) and drought stress (DS) libraries

	**CO**	**DS**
total tags	4719982	4804046
clean tags	4715625	4799759
clean tags copy number = 1	305492	319431
unique tags	328806	340187
unique tags copy number >5	87954	89327
unique tags copy number >10	40951	42909
unique tags copy number >20	20892	22972
unique tags copy number >50	9433	10896
unique tags copy number >100	5013	5672

### Depth of sampling

**Figure 2:**
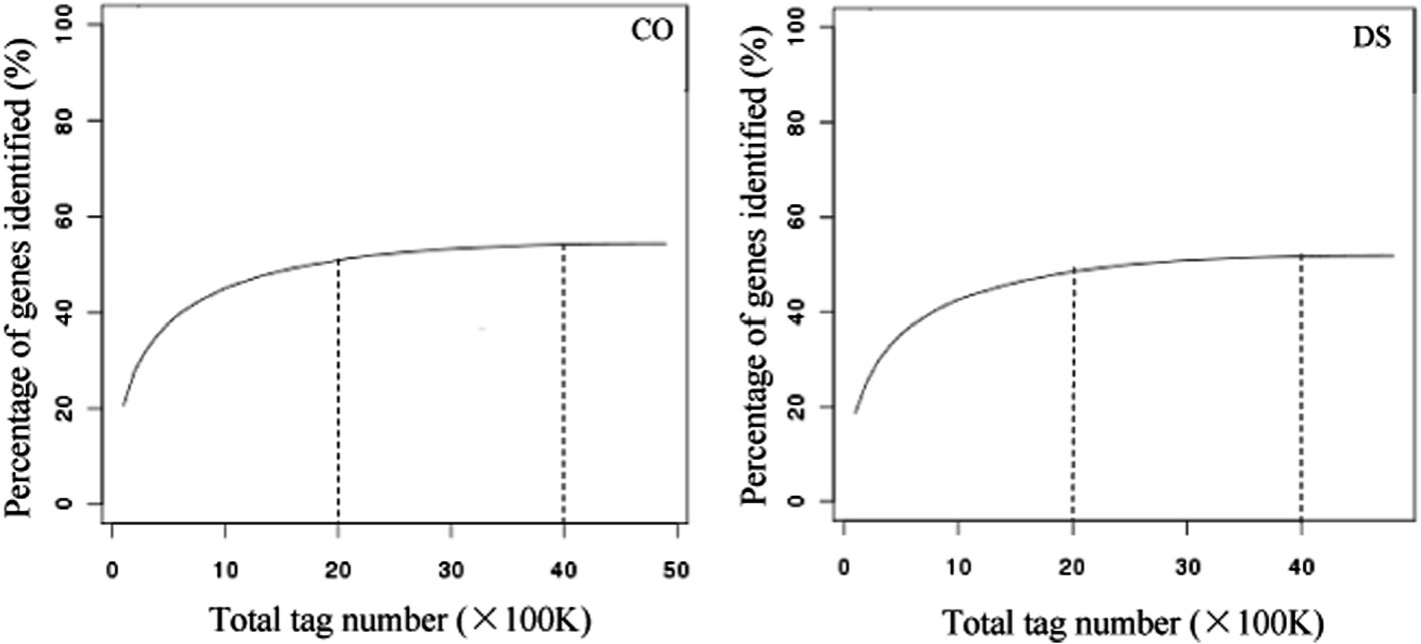
Saturation evaluations for the CO and DS libraries.

Saturation of the library is determined by checking the number of detected genes. Sequencing reaches saturation when no new genes are detected. The results showed that when sequencing amount reached 2 M or higher, there were few new genes detected in both libraries (Figure 2). The number of detected genes reached a plateau when 4 M tags were sequenced. No new genes were identified as the total tag number approached 4.7 M in CO library and 4.8 M in DS library. Therefore, the CO and DS libraries were sequenced to saturation, producing a full representation of the transcripts in the conditions tested.

### Annotation analysis of the unique tags

The ramie transcriptome had been *de novo* assembled and 43,990 unique genes were identified and annotated for their function [12]. In order to annotate the function of the tags sequenced in DS and CO libraries, the unique tags were aligned with these 43,990 unique genes using BLASTn. Tags with a complete match or one base pair mismatch were further considered to be used for estimating the expression level of gene. The results showed that 43,085 (12.67%) unique tags were matched to 23,912 (54.36%) in DS library and 39,143 (11.90%) unique tags were matched to 22,826 (51.89%) expression genes in CO library (Table 2).

**Table 2:** Annotation of illumina tags

**Library**	**Total tags**	**Unique tags**	**Match genes**
DS	1457659 (32.53%)	43085 (12.67%)	23912 (54.36%)
CO	1398438 (31.71%)	39143 (11.90%)	22826 (51.89%)

### Comparison of gene expression level between two libraries

**Figure 3:**
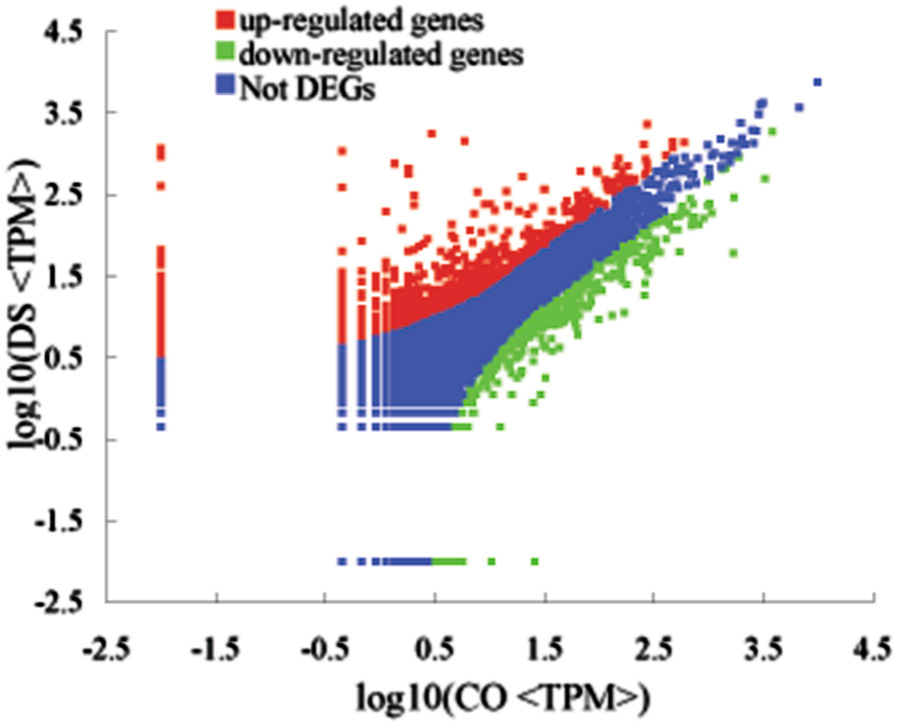
**Comparision of gene expression level between CO and DS libraries.** Red dots represent transcripts more prevalent in the DS library, green dots show those present at a lower frequency in the drought stress ramie and blue dots indicate transcripts that did not change significantly. The parameters “FDR ≤ 0.001” and “log2 Ratio ≥ 1” were used as the threshold to judge the significance of gene expression difference.

**Figure 4:**
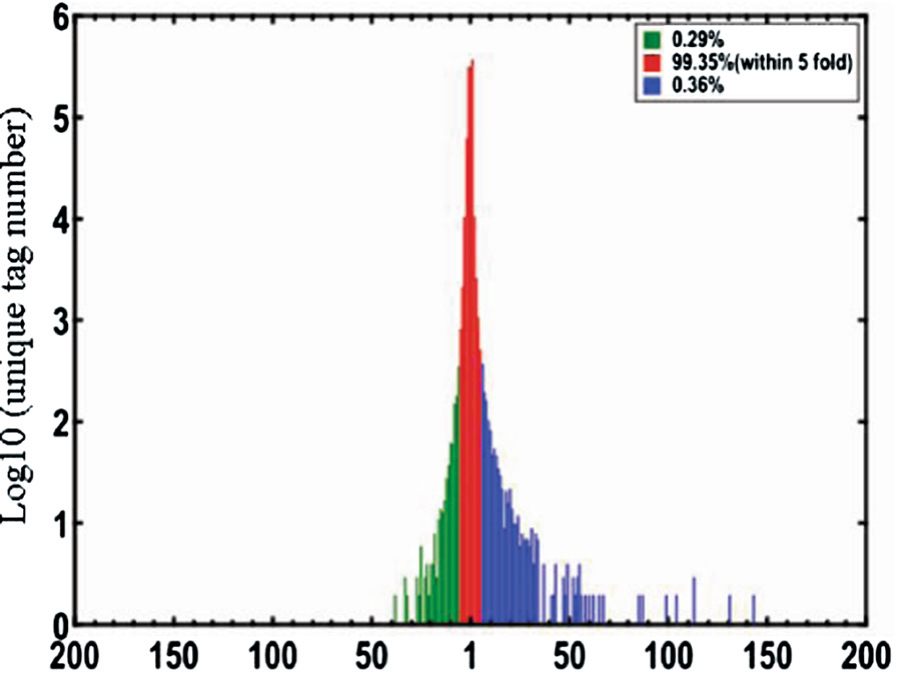
**Differentially expressed tags in DS tissue library.** The “x” axis represents fold-change of differentially expressed unique tags in the DS library. The “y” axis represents the number of unique tags (log10). Differentially accumulating unique tags within 5-fold difference between libraries are shown in the red region (99.35%). The blue (0.36%) and green (0.29%) regions represent unique tags that are up- and down regulated for more than 5 fold in the DS library, respectively.

Tag abundance appearing in library was used for estimating the expression level of gene mapped by tag. Differences of tag frequencies appearing in CO and DS libraries were used to determine the expression changes of genes in response to drought stress. The transcripts detected with at least two-fold differences in two libraries were shown in Figure 3 (FDR ≤ 0.001). The red dots (1,011) and green dots (505) respectively represent more and less abundant transcripts with more than two folds difference in DS library, designated as differentially expressed genes (DEGs, i.e. potential drought stress-responsive genes); while the blue dots represent transcripts with less than two-fold abundant difference between two libraries, which were designated as “no difference in expression”. In other words, a total of 1011 and 505 genes were probably up- and down- regulated under drought stress, respectively. The differentially expressed unique tags with more than five folds difference were shown in Figure 4. A total of 427 genes which were matched by about 0.36% total unique tags had a more than five-fold increase in expression abundance, and 123 genes matched by about 0.29% total unique tags had a decrease of abundance with more than 5 folds in the DS library, while the expression difference of 99.35% unique tags was within five-fold between two samples. Among 1516 potential drought stress-responsive genes, there were 157 genes up-regulated and 27 genes down-regulated with greater than hundred folds difference in DS library (Additional file 1) and 1258 genes showed significant similarity with known proteins in Nr database (Additional file 2).

### Potential pathway influenced by drought stress

The possible influence of drought stress on biological pathways was evaluated by enrichment analysis of DEGs. A total of 112 pathways were possibly affected by drought stress (Additional file 3). Pathways with Q value < 0.05 were significantly enriched by DEGs. Nine pathways may be severely influenced by drought stress for the significant enrichment of the DEGs (Q < 0.05) (Table 3). Among 9 enriched pathways, 5 pathways had more up-regulated DEGs; 3 pathways had more down-regulated DEGs; one pathway had a same number of up- and down-regulated potential stress-responsive genes. The Ribosome pathway enriched the most DEGs, followed by Starch and sucrose metabolism, Pentose and glucuronate interconversions, Phagosome, Other glycan degradation, Carbon fixation in photosynthetic organisms, Fructose and mannose metabolism, Ascorbate and aldarate metabolism and Riboflavin metabolism (Table 3).

**Table 3:** List of pathway significantly enriched in DEGs (Q < 0.05)

**Pathway**	**Background** **number**	**Regulated genes numbers**			**P value**	**Q value**	**Pathway ID**
		**Up-**	**Down-**	**Total**			
Ribosome	354	53	2	55	0.000	0.000	ko03010
Starch and sucrose metabolism	565	27	12	39	0.003	0.039	ko00500
Pentose and glucuronate interconversions	320	21	5	26	0.002	0.032	ko00040
Phagosome	238	21	1	22	0.001	0.017	ko04145
Other glycan degradation	123	17	2	19	0.000	0.000	ko00511
Carbon fixation in photosynthetic organisms	144	6	11	17	0.000	0.005	ko00710
Fructose and mannose metabolism	136	7	7	14	0.002	0.039	ko00051
Ascorbate and aldarate metabolism	102	5	6	11	0.005	0.049	ko00053
Riboflavin metabolism	48	1	6	7	0.004	0.049	ko00740

### Identification of drought stress-responsive TFs

Twenty-four potential drought stress-responsive transcription regulators were identified by Illumina tag-sequencing (Additional file 4). Twenty transcription factors (TFs) showed more and 4 TFs showed less abundance in DS library. Among 20 TFs up-regulated potentially, the unigene19721 encoding DELLA protein which is a key negative regulator in gibberellins (GAs) signal pathway, had more abundance with 335 folds difference in DS library. It is possible that the up-regulation of unigene19721 expression is responsible for the inhibition of the growth of ramie and the decrease of fiber yield under drought stress. Therefore, the expression level of unigene19721 and another eleven potential stress-responsive TFs were further analysis by qRT-PCR (Table 4).

**Table 4:** The function annotated of TFs validated by qRT-PCR

**Gene**	**Family**	**Function annotated**
Unigene4099	bHLH	UNE10-like transcription factor
Unigene8530	DOF	DOF domain class transcription factor
Unigene2022	C2H2L	C2H2L domain class transcription factor
Unigene957	AP2	AP2 domain class transcription factor
Unigene9044	NAC	NAC domain-containing protein 8, putative
Unigene13775	NAC	NAC domain-containing protein 7-like, putative
Unigene8373	NAC	NAC domain-containing protein 100-like, putative
Unigene19721	GRAS	DELLA protein, putative
Unigene565	HD-Zip	homeobox-leucine zipper protein ATHB-16-like, putative
Unigene1569	MYB	myb transcription factor
Unigene5955	ARF	auxin-responsive family protein, putative
Unigene19209	HD-Zip	transcription regulator, putative

**Figure 5:**
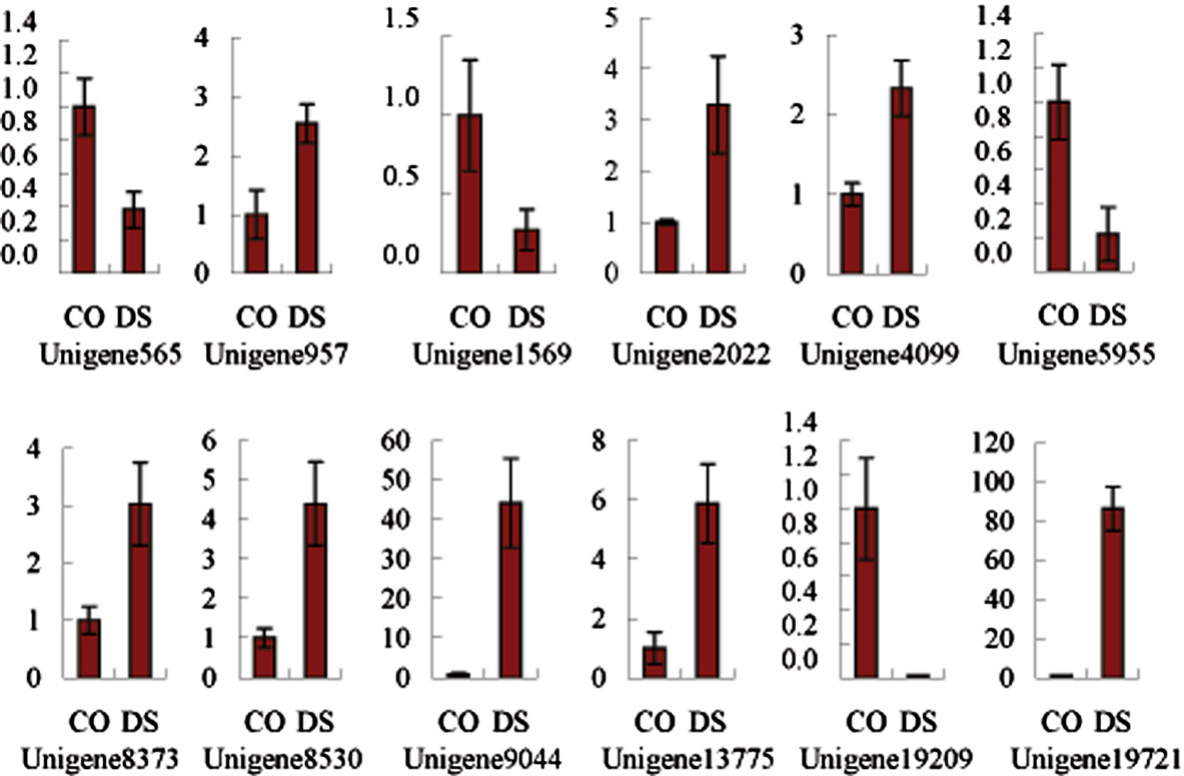
**qRT-PCR analysis of twelve differentially expressed TFs.** Data represent fold change of each DEG’s relative quantification in drought stress (DS) vs. control (CO) samples; the error bar represented the standard deviation.

The ramie actin gene (CL5463.Contig2) with a similar value of transcripts per million clean tags (TPM) in DS and CO libraries was selected as the endogenous control of qRT-PCR. The t-test showed that the qRT-PCR Ct value of actin in CO and DS ramie had no difference (P > 0.05). Thus, the actin expression did not have differences between the DS and CO ramie. The qRT-PCR result was presented in Figure 5. Six and three TFs were up- and down- regulated with 2~6 folds under water deficit condition. The unigene9044 and unigene19721 were up-regulated with more than 40 and 80 folds under drought stress, respectively; whereas unigene19209 was down-regulated with greater than 80 folds. These result suggested that all these 12 TFs were authentically drought stress-responsive TFs.

## Discussion

### Identification of 1516 potential drought stress-responsive genes by Illumina tag-sequencing

In China, almost 90% ramie distributes in Yangtze valley, which indicates that ramie has a poor eco-adaptability. The correlation between environment factors of ramie cultivation region and ramie fiber yield showed that the fiber yield severely depended on the rainfall of ramie growth region [13]. Ramie fiber extracted from stem bark is vegetative tissue and its yield is determined by the stem growth. In this study, severe inhibition of stem growth and significant decrease of fiber yield were observed in drought-stressed ramie. Except these morphological traits such as stem traits and fiber yield, a large number of physiological characters are easily influenced by water stress in ramie. Previous study showed that significant decreases in contents of chlorophyll *a*, carotenoid and endogenous GAs and relative water content, and increases in the activities of peroxidase, superoxide dismutase, and catalase and the contents of proline, malondialdehyde and soluble sugar were observed under drought stress [14]. Hence, sufficient water supply from growth environment is essential for ramie high production. However, in order to ensure the food security in China, the irrigable cultivated land was used to produce foodstuff and the ramie was mainly grown in un-irrigable dry land such as hill sloping land. Therefore, elucidating potential molecular responsive mechanism of ramie to water stress and improving its drought tolerance ability have important significance for ramie producing in China. In present study, on the basis of Illumina tag-sequencing technology, a total of 1516 potential drought stress-responsive genes were identified. The identification of these potential stress-responsive genes will be helpful for further understanding of ramie drought tolerance.

The Illumina sequencing technology is the next generation sequencing (NGS) which is a powerful tool utilized in many researching areas, including re-sequencing, micro-RNA expression profiling, DNA methylation, *de novo* transcriptome sequencing and whole sequencing [15-21], especially in the analysis of whole-genome expression profiling [22-25]. In current study, the NGS was used to identify the potential drought stress-responsive genes based on principle that tag frequencies of given gene can be used to estimate its transcript abundance. Theoretically, tags should be generated by NlaIII from the 3′-most ends of transcripts. Other tags may also be generated because of incomplete enzyme digestion in practice. Since only one tag could be generated in each transcript from any NlaIII site in a cDNA, one tag represented a transcript of a given gene. In other word, the total of all NlaIII tags’ copy number in a gene represented the transcript abundance of this gene in library. For alternative splicing, it was possible that there were some genes spliced as multiple transcripts. In our study, when one tag matched to multiple transcripts, this tag would not be used to estimate the expression level. Out of 43,990 reference genes [12], about 54.4% and 51.9% genes were matched by tags of DS and CO library. Several potential reasons were responsible for the residual genes which were not matched by tags. First, there were about 20% genes without restriction enzyme cutting site of NlaIII, which led to a fact that these 20% genes could not generate their tag. Second, the reference transcriptome used in this study were sequenced based on the RNA of several growth stages, including seedling, vigorous vegetative growth stage and fiber ripeness stage [12], while the RNA used to construct library in this study was only extracted from the tissue of 30-day-old ramie. Probably, some genes which only express in seedling and fiber ripeness stage will not appear in the library constructed in this study. Moreover, a large number of ESTs in reference transcriptome are partial sequence of genes. Hence, of these 43,990 ESTs in reference transcriptome, it is likely that several ESTs are sequenced from a common gene which only generates a type of tag by NlaIII from the 3′-most ends of gene. In other words, only one of several ESTs sequenced from a gene can be aligned with the tag of this gene and the others can not be annotated by tags, which is another major reason for the presence of about 40% genes un-annotated.

### Transcription factors responding to drought stress in ramie

Transcriptome analyses using microarray technology, together with conventional approaches, have revealed that dozens of transcription factors (TFs) are involved in the plant response to drought stress [4,9,10]. Most of these TFs fall into several large TF families, such as AP2/ERF, bZIP, NAC, MYB, MYC, Cys2His2 zinc-finger and WRKY. The expression of TFs regulates the expression of downstream target genes which are involved in the drought stress response and tolerance. Up to now, hundreds of TFs were validated in its ability of drought tolerance by further study. Taking NAC TFs as an example, scores of NAC TFs in rice, Arabidopsis, wheat, maize and so on were found to respond to abiotic and biotic stress and over-expression of these TFs can improve the drought tolerance ability of transgenic plant [26-31]. In this study, a total of 24 TFs involving in several families such as NAC, MYB, HD-Zip, AP2/ERF and so on were found that they are probably differential expression (20 TFs up-regulated and 4 TFs down-regulated) between well-watered and drought-stressed ramie by Illumina tag-sequencing. Twelve TFs (3 NAC TFs, 2 HD-Zip TFs, 1 bHLH TF, 1 DOF TF, 1 C2H2L TF, 1 MYB TF, 1 AP2 TF, 1 ARF TF and 1 GRAS TF) were chosen for further expression analysis in well-watered and drought-stressed ramie by qRT-PCR and the result validated that all these 12 TFs were drought stress-responsive TFs.

### Up-regulation of unigene19721 expression is probably responsible for the inhibition of the growth of drought-stressed ramie

Under drought condition, a major morphological characteristic of plants is dwarfism, which is considered as an adaptive change of plants to help them avoid high energy costs under unfavorable conditions [32]. Opposite to drought inhibiting the growth, GAs can stimulate stem elongation and promote the growth of plants [33]. There is a potential crosstalk between drought stress signal and GAs signal resulting in antagonic interaction to regulate the plant growth. DELLA protein is a negative regulator of GAs signal pathway and can inhibit the growth of plants. GAs signal can induce the destruction of DELLA protein and then relieve the repression function of DELLA [33]. Previous studies showed that DELLA protein can respond to abiotic and biotic stress [34], and the accumulation of DELLA protein markedly improves the ability of stress tolerance [35-37]. Therefore, DELLA protein not only functions as negative regulator to repress the growth of plants but also enhances the ability of stress tolerance of plants. In this study, a DELLA protein encoded gene, unigene19721, was found up-regulated expression with 335 folds under drought stress. The up-regulated expression of this gene in drought-stressed ramie was further confirmed by qRT-PCR. Probably, ramie increases its DELLA protein to enhance drought tolerance ability by up-regulating the expression of unigene19721 under water deficit, whereas the increase of DELLA protein leads to a corresponding inhibition of stem elongation and decrease of fiber yield in ramie.

## Conclusion

In this study, a total of 1516 potential drought stress-responsive genes including 24 TFs were identified in ramie. Twelve TFs were further validated to involve in the response of drought stress by qRT-PCR. Among the 12 stress-responsive TFs, the unigene19721 encoding the DELLA protein which is a key negative regulator in gibberellins signal pathway was markedly up-regulated, which is probably responsible for the inhibition of the growth of ramie under drought stress. The identification of these candidate TFs which may contribute to drought tolerance in ramie will be helpful for further improving ramie drought tolerance ability.

## Methods

### Plant material, treatment of water stress and RNA extraction

Elite ramie variety Zhongzhu 1 was used in this study. Zhongzhu 1 is an elite variety with characteristics of high yield, good fiber quality and strong drought resistance and it has the largest growth area in China during recent years. The cuttage seedlings of Zhongzhu 1 were transplanted to pot in May 2011. In April 2012, the potted 30-day-old ramies were transferred to a movable rain-off shelter and were parted into two groups. One group (CO) where the ramie was grown under well water condition by daily watering was used as control, and the other group (DS) was treated with drought stress by controlling the relative water content of soil at a level of no more than 35%. Each group was planted with three replicates. After seven days the ramie suffering from drought stress, the CO and DS ramie tissues of three replicates including leaf, root, stem bast, stem xylem and stem shoot were individually collected. The sampled tissues were immediately frozen in liquid nitrogen and stored at −80° until use. The same tissue sample of three replicates of each group was mixed to extract RNA. Total RNAs of two treatment ramie were extracted from each tissue sample using TRIzol reagent (Transgene Company, Illkirch Graffenstaden Cedex, France) according to the manufacturer’s protocol. The RNA was equally pooled from the five tissues for cDNA library preparation.

### Preparation of digital expression libraries

Sequence tag preparation was done with the Digital Gene Expression Tag Profiling Kit (Illumina Inc; San Diego, CA, USA) according to the manufacturer’s protocol. Six micrograms of total RNA for CO and DS ramie was individually purified using biotin-Oligo (dT) magnetic bead adsorption. First- and second-strand cDNA synthesis was performed after the RNA was bound to the beads. While on the beads, double strand cDNA was digested with NlaIII endonuclease to produce a bead-bound cDNA fragment containing sequence from the 3′-most CATG to the poly (A)-tail. These 3′ cDNA fragments were purified by using magnetic bead precipitation and the Illumina adapter 1 (GEX adapter 1) was added to new 5′ end. The junction of Illumina adapter 1 and CATG site was recognized by MmeI, which is a Type I endonuclease (with separated recognition sites and digestion sites). The enzyme cuts 17 bp downstream of the CATG site, producing 17 bp cDNA sequence tags with adapter 1. After removing 3′ fragments with magnetic bead precipitation, the Illumina adapter 2 (GEX adapter 2) was ligated to 3′ end of the cDNA tag. These cDNA fragments represented the tag library.

### Illumina sequencing

Illumina sequencing using the HiSeq^TM^ 2000 platform was performed at Beijing Genomics Institute (BGI)-Shenzhen, Shenzhen, China (http://www.genomics.cn/) with the method of sequencing by synthesis. Briefly, PCR amplification with 15 cycles using Phusion polymerase (Finnzymes, Espoo, Finland) was performed with primers complementary to the adapter sequences to enrich the samples for the desired fragments. The resulting 105 base strips were purified by 6% TBE PAGE Gel electrophoresis. These strips were then digested, and the single chain molecules were fixed onto the Illumina Sequencing Chip (flow cell). Each molecule grew into a single-molecule cluster sequencing template through in situ amplification. Four color-labeled nucleotides were added, and sequencing was performed with the method of sequencing by synthesis. Image analysis and base calling were performed by using the Illumina Pipeline, and cDNA sequence tags were revealed after purity filtering. The tags passing initial quality tests were sorted and counted. Each tunnel generates millions of raw reads with sequencing length of 49 bp (target tags plus 3′adaptor). Each molecule in the library represented a single tag derived from a single transcript.

### Gene annotation

“Clean Tags” were obtained by filtering off adaptor-only tags and low-quality tags (containing ambiguous bases). Comparison of the Clean Tags sequences with ramie transcriptome sequence [12] by BLASTN was carried out. All clean tags were annotated based on ramie reference genes. The number of annotated clean tags for each gene was calculated and then normalized to TPM (number of transcripts per million clean tags) [24,25].

### Identification of differentially expressed genes (DEGs)

A rigorous algorithm to identify differentially expressed genes between two samples was developed [38]. P value was used to test differential transcript accumulation. In the formula below the total clean tag number of the CO library is noted as N1, and total clean tag number of DS library as N2; gene A holds x tags in CO and y tags in DS library. The probability of gene A expressed equally between two samples can be calculated with:

$$P\left(y|x\right)={\left(\frac{N2}{N1}\right)}^{y}\frac{\left(x+y\right)!}{x!y!{\left(1+\frac{N2}{N1}\right)}^{\left(x+y+1\right)}}$$

FDR (False Discovery Rate) was applied to determine the threshold of P Value in multiple tests and analyses [39]. An “FDR < 0.001 and the absolute value of log2-Ratio ≥ 1” was used as the threshold to judge the significance of gene expression difference.

### Real-time quantitative PCR (qRT-PCR) analysis

The CO and DS ramie were used for qRT-PCR analysis. Entire plants of six individuals (three CO plants and three DS plants) were individually sampled. The sampled tissues were immediately frozen in liquid nitrogen and used to extract RNA. For each sample, first-strand cDNAs were reverse-transcribed from RNAs treated with DNase I (Fermentas, Canada) by using M-MuLV Reverse Transcriptase (Fermentas, Canada) according to the manufacturer’s instructions. qRT-PCR was performed using an optical 96-well plate with an iQ5 multicolor real time PCR system (Bio-RAD, USA). Each reaction contained 1 μL of cDNA template, 10 nM gene-specific primers, 10 μL of SYBR Premix Ex Taq, and 0.4 μL of ROX Reference Dye (FINNZYMES, Finland) in a final volume of 20 μL. The ramie actin gene was selected as the endogenous control. The primer sequence of DEGs and actin gene were listed in Additional file 5. The thermal cycle used was as follows: 95°C for 15 min, followed by 40 cycles of 95°C for 10 s, 55°C for 20s and 72°C for 30 s. qRT-PCR was performed in triplicate for each sample. Relative expression levels were determined as described previously [40].

### Pathway enrichment analysis of DEGs

Pathway enrichment analysis based on KEGG (Kyoto Encyclopedia of Genes and Genomes pathway database http://www.genome.jp/kegg) was used to identify significantly enriched metabolic pathways or signal transduction pathways in differentially expressed genes comparing with the whole genome background. The calculating formula is:

$$p=1-\sum_{i=0}^{m-i}\frac{\left(\begin{array}{c} M\\ i \end{array}\right)\left(\begin{array}{c} N-M\\ n-i \end{array}\right)}{\left(\begin{array}{c} N\\ n \end{array}\right)},$$

where N is the number of all genes that with KEGG annotation, n is the number of DEGs in N, M is the number of all genes annotated to specific pathways, and m is number of DEGs in M. Q value was used for determining the threshold of P Value in multiple test and analysis [41]. Pathways with Q value < 0.05 are significantly enriched in DEGs.

### Availability of supporting data

The raw data (tag sequences, counts and TPM value) has been deposited at the Gene Expression Omnibus [NCBI GEO] with accession number GSE46253, [http://www.ncbi.nlm.nih.gov/geo/query/acc.cgi?acc=GSE46253].

## Abbreviations

DS: Drought stress; CO: Control; DEG: Differentially expressed gene; qRT-PCR: Quantitative real-time polymerase chain reaction; TF: Transcription factor; TPM: Transcripts per million clean tags; KEGG: Kyoto Encyclopedia of Genes and Genomes pathway database.

## Competing interests

The authors have declared that no competing interests exist.

## Authors’ contributions

LT and TS conceived and designed the experiment. LT and ZS performed the experiment. TQ and YY helped to prepare the reagents and materials. LT carried out the data analysis and wrote the manuscript. All authors read and approved the final manuscript.

## Supplementary Material

Additional file 1 DEGs with more than 100 folds between DS and CO libraries. Click here for file

Additional file 2 DEGs between DS and CO libraries. Click here for file

Additional file 3 Potential pathways affected by drought stress. Click here for file

Additional file 4 Potential drought stress-responsive transcription factors. Click here for file

Additional file 5 Primers of genes validated by qRT-PCR. Click here for file

## References

[B1] Bray E Plant responses to water deficit Trends Plant Sci 1997 2 48 54

[B2] Zhang Q Strategies for developing Green Super Rice Proc Natl Acad Sci U S A 2007 104 16402 16409 10.1073/pnas.0708013104 17923667 PMC2034246

[B3] Franca M Prados L Lemos-Filho J Ranieri B Vale F Morphophysiological differences in leaves of Lavoisiera campos-portoana (Melastomataceae) enhance higher drought tolerance in water shortage events J Plant Res 2012 125 85 92 10.1007/s10265-011-0416-z 21400250

[B4] Yamaguchi-Shinozaki K Shinozaki K Transcriptional regulatory networks in cellular responses and tolerance to dehydration and cold stresses Annu Rev Plant Biol 2006 57 781 803 10.1146/annurev.arplant.57.032905.105444 16669782

[B5] Urano K Maruyama K Ogata Y Morishita Y Takeda M Sakurai N Suzuki H Saito K Shibata D Kobayashi M Characterization of the ABA regulated global responses to dehydration in Arabidopsis by metabolomics Plant J 2009 57 1065 1078 10.1111/j.1365-313X.2008.03748.x 19036030

[B6] Rabbani M Maruyama K Abe H Khan M Katsura K Ito Y Yoshiwara K Seki M Shinozaki K Yamaguchi-Shinozaki K Monitoring expression profiles of rice genes under cold, drought, and high-salinity stresses and abscisic acid application using cDNA microarray and RNA Gel-Blot analyses Plant Physiol 2003 133 1755 1767 10.1104/pp.103.025742 14645724 PMC300730

[B7] Zheng J Fu J Gou M Huai J Liu Y Jian M Huang Q Guo X Dong Z Wang H Genome-wide transcriptome analysis of two maize inbred lines under drought stress Plant Mol Biol 2010 72 407 421 10.1007/s11103-009-9579-6 19953304

[B8] Aprile A Mastrangelo A Leonardis A Galiba G Roncaglia E Ferrari F Bellis L Turchi L Giuliano G Cattivelli L Transcriptional profiling in response to terminal drought stress reveals differential responses along the wheat genome BMC Genomics 2009 10 279 10.1186/1471-2164-10-279 19552804 PMC2713995

[B9] Nakashima K Ito Y Yamaguchi-Shinozaki K Transcriptional regulatory networks in response to abiotic stresses in Arabidopsis and grasses Plant Physiol 2009 149 88 95 10.1104/pp.108.129791 19126699 PMC2613698

[B10] Fujita Y Fujita M Shinozaki K Yamaguchi-Shinozaki K ABA-mediated transcriptional regulation in response to osmotic stress in plants J Plant Res 2011 124 509 525 10.1007/s10265-011-0412-3 21416314

[B11] Liu F Liu Q Liang X Huang H Zhang S Morphological, anatomical, and physiological assessment of ramie [Boehmeria Nivea (L.) Gaud.] tolerance to soil drought Genetic Resources and Crop Evolution 2005 52 497 506 10.1007/s10722-004-7071-3

[B12] Liu T Zhu S Tang Q Chen P Yu Y Tang S *De novo* assembly and characterization of transcriptome using Illumina paired-end sequencing and identification of CesA gene in ramie (*Boehmeria nivea L.Gaud*) BMC Genomics 2013 14 125 10.1186/1471-2164-14-125 23442184 PMC3610122

[B13] Liu T Tang Q Zhu S Tang S Analysis of climatic factors causing yield difference in ramie among different eco-regions of Yalley valley Agricultural Science & Technology 2011 12 745 75024023804

[B14] Liu T Zhu S Fu L Yu Y Tang Q Tang S Morphological and physiological changes of ramie (*Boehmeria nivea* L. Gaud) in response to drought stress and GA_3_ treatment Russian Journal of Plant Physiology 2013 60 749 755

[B15] Meyer E Aglyamova GV Wang S Buchanan-Carter J Abrego D Colbourne J Willis B Matz M Sequencing and de novo analysis of a coral larval transcriptome using 454 GSFlx BMC Genomics 2009 10 219 10.1186/1471-2164-10-219 19435504 PMC2689275

[B16] Wang J Wang W Li R Li Y Tian G Goodman L Fan W Zhang J Li J Zhang J The diploid genome sequence of an Asian individual Nature 2008 456 60 65 10.1038/nature07484 18987735 PMC2716080

[B17] Huang Y Huang T Wang L Profiling DNA methylomes from microarray to genome-scale sequencing Technol Cancer Res Treat 2010 9 139 147 20218736 10.1177/153303461000900203 PMC3011833

[B18] Nobuta K McCormick K Nakano M Meyers BC Bioinformatics analysis of small RNAs in plants using next generation sequencing technologies Methods Mol Biol 2010 592 89 106 10.1007/978-1-60327-005-2_7 19802591

[B19] Barakat A DiLoreto D Zhang Y Smith C Baier K Powell W Wheeler N Sederoff R Carlson J Comparison of the transcriptomes of American chestnut (Castanea dentata) and Chinese chestnut (Castanea mollissima) in response to the chestnut blight infection BMC Plant Biol 2009 9 51 10.1186/1471-2229-9-51 19426529 PMC2688492

[B20] Fahlgren N Howell M Kasschau K Chapman E Sullivan C Cumbie J Givan S Law T Grant S Dang J High-Throughput Sequencing of Arabidopsis microRNAs: Evidence for Frequent Birth and Death of MIRNA Genes PLoS ONE 2007 2 e29 10.1371/journal.pone.0000219 PMC1790633 17299599

[B21] Wang Z Fang B Chen J Zhang X Luo Z Huang L Chen X Li Y De novo assembly and characterization of root transcriptome using Illumina paired-end sequencing and development of cSSR markers in sweetpotato (Ipomoea batatas) BMC Genomics 2010 11 726 10.1186/1471-2164-11-726 21182800 PMC3016421

[B22] Wu J Zhang Y Zhang H Huang H Folta K Lu J Whole genome wide expression profiles of Vitis amurensis grape responding to downy mildew by using Solexa sequencing technology BMC Plant Biol 2010 10 234 10.1186/1471-2229-10-234 21029438 PMC3017854

[B23] Eveland A Satoh-Nagasawa N Goldshmidt A Meyer S Beatty M Sakai H Ware D Jackson D Digital gene expression signatures for maize development Plant Physiol 2010 154 1024 1039 10.1104/pp.110.159673 20833728 PMC2971585

[B24] Morrissy A Morin R Delaney A Zeng T McDonald H Jones S Zhao Y Hirst M Marra M Next-generation tag sequencing for cancer gene expression profiling Genome Res 2009 19 1825 1835 10.1101/gr.094482.109 19541910 PMC2765282

[B25] 't Hoen P Ariyurek Y Thygesen H Vreugdenhil E Vossen R Menezes R Boer J Ommen G Dunnen J Deep sequencing-based expression analysis shows major advances in robustness, resolution and inter-lab portability over five microarray platforms Nucleic Acids Res 2008 36 e141 10.1093/nar/gkn705 18927111 PMC2588528

[B26] Wu Y Deng Z Lai J Zhang Y Yang C Yin B Zhao Q Zhang L Li Y Yang C Dual function of Arabidopsis ATAF1 in abiotic and biotic stress responses Cell Res 2009 19 1279 1290 10.1038/cr.2009.108 19752887

[B27] Tran LS Nakashima K Sakuma Y Simpson S Fujita Y Maruyama K Fujita M Seki M Shinozaki K Yamaguchi-Shinozaki K Isolation and functional analysis of Arabidopsis stress inducible NAC transcription factors that bind to a drought responsive cis-element in the early responsive to dehydration stress 1 promoter Plant Cell 2004 16 2481 2498 10.1105/tpc.104.022699 15319476 PMC520947

[B28] Hu H Dai M Yao J Xiao B Li X Zhang Q Xiong L Overexpressing a NAM, ATAF, and CUC (NAC) transcription factor enhances drought resistance and salt tolerance in rice Proc Natl Acad Sci U S A 2006 103 12987 12992 10.1073/pnas.0604882103 16924117 PMC1559740

[B29] Jeong J Kim Y Baek K Jung H Ha S Choi Y Kim M Reuzeau C Kim J Root-specific expression of OsNAC10 improves drought tolerance and grain yield in rice under field drought conditions Plant Physiol 2010 153 185 197 10.1104/pp.110.154773 20335401 PMC2862432

[B30] Nakashima K Tran L Nguyen D Fujita M Maruyama K Todaka D Ito Y Hayashi N Shinozaki K Yamaguchi-Shinozaki K Functional analysis of a NAC type transcription factor OsNAC6 involved in abiotic and biotic stress responsive gene expression in rice Plant J 2007 51 617 630 10.1111/j.1365-313X.2007.03168.x 17587305

[B31] Zheng X Chen B Lu G Han B Overexpression of a NAC transcription factor enhances rice drought and salt tolerance Biochem Biophys Res Commun 2009 379 985 989 10.1016/j.bbrc.2008.12.163 19135985

[B32] Li J Sima W Ouyang B Wang T Ziaf K Luo Z Liu L Li H Chen M Huang Y Tomato *SlDREB* gene restricts leaf expansion and internode elongation by downregulating key genes for gibberellin biosynthese J Exp Bot 2012 63 6407 6420 10.1093/jxb/ers295 23077200 PMC3504492

[B33] Hauvermale A Ariizumi T Steber C Gibberellin signaling: a theme and variations on DELLA repression Plant Physiol 2012 160 83 92 10.1104/pp.112.200956 22843665 PMC3440232

[B34] Sun T The molecular mechanism and evolution of the review GA–GID1–DELLA signaling module in plants Curr Biol 2011 21 338 345 10.1016/j.cub.2011.01.036 21549956

[B35] Achard P Cheng H Grauwe L Decat J Schoutteten H Moritz T Straeten D Peng JR Harberd NP Integration of plant responses to environmentally activated phytohormonal signals Science 2006 311 91 94 10.1126/science.1118642 16400150

[B36] Achard P Renou JP Berthomé R Harberd NP Genschik P Plant DELLAs restrain growth and promote survival of adversity by reducing the levels of reactive oxygen species Curr Biol 2008 18 656 660 10.1016/j.cub.2008.04.034 18450450

[B37] Jiang C Gao X Liao L Harberd N Fu X Phosphate starvation root architecture and anthocyanin accumulation responses are modulated by the gibberellin-DELLA signaling pathway in Arabidopsis Plant Physiol 2007 145 1460 1470 10.1104/pp.107.103788 17932308 PMC2151698

[B38] Audic S Claverie JM The significance of digital gene expression profiles Genome Res 1997 7 986 995 9331369 10.1101/gr.7.10.986

[B39] Benjamini Y Drai D Elmer G Kafkafi N Golani I Controlling the false discovery rate in behavior genetics research Behav Brain Res 2001 125 279 284 10.1016/S0166-4328(01)00297-2 11682119

[B40] Livak KJ Schmittgen TD Analysis of relative gene expression data using real-time quantitative PCR and the 2(−Delta Delta C(T)) Method Methods 2001 25 402 408 10.1006/meth.2001.1262 11846609

[B41] Benjamini Y Hochberg Y Controlling the false discovery rate: a practical and powerful approach to multiple testing J Roy Stat Soc B 1995 57 289 300

