# Supplementary material for: Identification of drought stress-responsive transcription factors in ramie (Boehmeria nivea L. Gaud)
Source: BMC Plant Biol. 2013 Sep 10;13:130. doi: 10.1186/1471-2229-13-130 (PMC3846573; doi:10.1186/1471-2229-13-130)
Supplement: Additional file 1 — DEGs with more than 100 folds between DS and CO libraries. [file 1471-2229-13-130-S1.doc]

| Gene | Fold | Annotation by blast nr |
| --- | --- | --- |
| Unigene1165 | 116576 | - |
| Unigene556 | 93185 | putative allergen Rub i 3 [Rubus idaeus] |
| Unigene2891 | 40577 | - |
| CL2935.Contig1 | 6584 | hypothetical protein VITISV_034041 [Vitis vinifera] |
| Unigene81 | 5312 | protein IQ-DOMAIN 1-like [Glycine max] |
| Unigene3184 | 5000 | - |
| Unigene771 | 4776 | - |
| CL1409.Contig1 | 4330 | unnamed protein product [Vitis vinifera] |
| Unigene7362 | 3214 | actin-depolymerizing factor 5 [Vitis vinifera] |
| Unigene13227 | 3125 | unnamed protein product [Vitis vinifera] |
| Unigene974 | 2589 | opper ion binding protein, putative [Ricinus communis] |
| Unigene5549 | 2455 | - |
| Unigene11259 | 2415 | - |
| Unigene15119 | 2210 | predicted protein [Populus trichocarpa] |
| Unigene479 | 2187 | - |
| Unigene1457 | 2053 | peroxidase [Ficus carica] |
| Unigene8354 | 2009 | Endo-1,4-beta-xylanase C precursor, putative [Ricinus communis] |
| Unigene12162 | 1920 | - |
| Unigene3698 | 1808 | predicted protein [Populus trichocarpa] |
| CL1611.Contig1 | 1763 | PGIP (chloroplast) [Morus alba var. multicaulis] |
| Unigene24708 | 1719 | - |
| Unigene7405 | 1652 | - |
| Unigene7760 | 1652 | - |
| Unigene4975 | 1384 | Pectin acetylesterase [Medicago truncatula] |
| Unigene16435 | 1295 | predicted protein [Populus trichocarpa] |
| CL4240.Contig1 | 1272 | predicted protein [Populus trichocarpa] |
| Unigene24583 | 1138 | - |
| Unigene4728 | 1138 | predicted protein [Populus trichocarpa] |
| Unigene3092 | 1094 | Protein P21, putative [Ricinus communis] |
| Unigene11207 | 1071 | unnamed protein product [Vitis vinifera] |
| CL2140.Contig2 | 1071 | polyphenol oxidase [Canarium album] |
| Unigene6397 | 1027 | probable polygalacturonase-like [Glycine max] |
| Unigene3475 | 1004 | fatty acyl-CoA reductase 3-like isoform 1 [Glycine max] |
| Unigene3309 | 982 | - |
| Unigene10096 | 960 | conserved hypothetical protein [Ricinus communis] |
| Unigene23465 | 893 | Microtubule-associated protein TORTIFOLIA1, putative [Ricinus communis] |
| Unigene770 | 867 | copper ion binding protein, putative [Ricinus communis] |
| Unigene2108 | 848 | - |
| CL3079.Contig1 | 848 | predicted protein [Populus trichocarpa] |
| Unigene11077 | 848 | Chromosome-associated kinesin KIF4A, putative [Ricinus communis] |
| Unigene29521 | 826 | - |
| Unigene9043 | 804 | uncharacterized protein LOC100241687 [Vitis vinifera] |
| CL5416.Contig1 | 804 | Pectinesterase-2 precursor, putative [Ricinus communis] |
| Unigene10899 | 781 | uncharacterized protein LOC100267070 [Vitis vinifera] |
| Unigene15293 | 781 | unnamed protein product [Vitis vinifera] |
| Unigene3773 | 759 | S-like ribonuclease [Prunus dulcis] |
| CL2931.Contig1 | 737 | unnamed protein product [Vitis vinifera] |
| Unigene2150 | 714 | predicted protein [Populus trichocarpa] |
| Unigene22175 | 714 | - |
| Unigene21158 | 714 | - |
| Unigene6641 | 692 | predicted protein [Populus trichocarpa] |
| Unigene22498 | 670 | - |
| Unigene13683 | 625 | - |
| Unigene13291 | 603 | hypothetical protein RCOM_1749890 [Ricinus communis] |
| Unigene15783 | 603 | predicted protein [Populus trichocarpa] |
| Unigene929 | 597 | VMP4 protein [Volvox carteri f. nagariensis] |
| CL194.Contig3 | 580 | polyphenol oxidase 1 [Fragaria pentaphylla] |
| Unigene10364 | 580 | predicted protein [Populus trichocarpa] |
| Unigene6462 | 580 | aspartic proteinase nepenthesin-2-like [Vitis vinifera] |
| Unigene541 | 559 | bifunctional inhibitor/lipid transfer protein/seed storage protein-like protein [Arabidopsis thaliana] |
| Unigene13430 | 558 | hypothetical protein RCOM_1313640 [Ricinus communis] |
| Unigene4677 | 558 | delta 1-pyrroline-5-carboxylate synthetase [Gossypium arboreum] |
| Unigene1035 | 558 | unnamed protein product [Vitis vinifera] |
| Unigene750 | 536 | unknown [Populus trichocarpa] |
| Unigene28124 | 513 | - |
| Unigene19333 | 513 | predicted protein [Populus trichocarpa] |
| Unigene23405 | 513 | probable pectinesterase/pectinesterase inhibitor 7-like [Glycine max] |
| Unigene3968 | 513 | ribulose 1,5-bisphosphate carboxylase small subunit [Malus x domestica x Pyrus communis] |
| Unigene3034 | 491 | uncharacterized protein LOC100797266 [Glycine max] |
| Unigene6927 | 491 | - |
| Unigene3632 | 491 | conserved hypothetical protein [Ricinus communis] |
| Unigene1598 | 491 | hypothetical protein VITISV_014848 [Vitis vinifera] |
| Unigene23007 | 469 | unnamed protein product [Vitis vinifera] |
| Unigene5207 | 469 | uncharacterized protein LOC100265200 [Vitis vinifera] |
| Unigene7268 | 469 | predicted protein [Populus trichocarpa] |
| Unigene24673 | 446 | - |
| Unigene8975 | 446 | uncharacterized protein LOC100243168 [Vitis vinifera] |
| Unigene1560 | 424 | gibberellin-regulated protein 4 [Vitis vinifera] |
| Unigene11636 | 424 | predicted protein [Populus trichocarpa] |
| CL4183.Contig2 | 424 | aspartic proteinase nepenthesin-2 [Vitis vinifera] |
| Unigene4269 | 424 | histone 2 [Populus trichocarpa] |
| Unigene3594 | 424 | BRASSINOSTEROID INSENSITIVE 1-associated receptor kinase 1 precursor, putative [Ricinus communis] |
| Unigene9188 | 402 | transferase, transferring glycosyl groups, putative [Ricinus communis] |
| Unigene8336 | 402 | hypothetical protein VITISV_007009 [Vitis vinifera] |
| Unigene9656 | 402 | - |
| Unigene20764 | 402 | predicted protein [Populus trichocarpa] |
| Unigene5292 | 402 | Serine/threonine-protein kinase PBS1, putative [Ricinus communis] |
| Unigene13188 | 379 | ATP binding protein, putative [Ricinus communis] |
| CL5168.Contig1 | 379 | Early nodulin 55-2 precursor, putative [Ricinus communis] |
| Unigene11654 | 379 | predicted protein [Populus trichocarpa] |
| Unigene5016 | 379 | beta tubulin [Setaria viridis] |
| Unigene7633 | 379 | CYP [Gossypium hirsutum] |
| Unigene11640 | 379 | squamosa promoter-binding-like protein 8-like [Glycine max] |
| Unigene564 | 363 | hypothetical protein VITISV_037748 [Vitis vinifera] |
| Unigene2547 | 357 | homeobox protein knotted-1-like 7-like [Glycine max] |
| Unigene19061 | 357 | - |
| Unigene14031 | 357 | unnamed protein product [Vitis vinifera] |
| Unigene11691 | 357 | Hippocampus abundant transcript-like protein [Medicago truncatula] |
| Unigene23861 | 357 | predicted protein [Populus trichocarpa] |
| CL203.Contig2 | 357 | ransferase, transferring glycosyl groups, putative [Ricinus communis] |
| Unigene10753 | 357 | - |
| Unigene13796 | 335 | - |
| Unigene24777 | 335 | PREDICTED: uncharacterized protein LOC100255813 [Vitis vinifera] |
| Unigene9044 | 335 | NAC domain-containing protein 8 [Vitis vinifera] |
| CL393.Contig2 | 335 | unnamed protein product [Vitis vinifera] |
| Unigene344 | 335 | predicted protein [Populus trichocarpa] |
| Unigene17064 | 335 | - |
| Unigene12993 | 335 | - |
| Unigene10725 | 335 | Bipolar kinesin KRP-130, putative [Ricinus communis] |
| Unigene19721 | 335 | DELLA protein RGL1, putative [Ricinus communis] |
| CL78.Contig1 | 335 | long chain acyl-CoA synthetase 2-like [Glycine max] |
| CL844.Contig2 | 335 | Potassium transporter, putative [Ricinus communis] |
| Unigene24459 | 335 | hypothetical protein VITISV_041326 [Vitis vinifera] |
| Unigene26521 | 335 | - |
| Unigene3606 | 335 | - |
| Unigene10384 | 335 | predicted protein [Populus trichocarpa] |
| Unigene635 | 313 | Non-specific lipid-transfer protein 3 |
| Unigene19448 | 312 | PI-PLC X domain-containing protein At5g67130-like [Glycine max] |
| Unigene23402 | 312 | - |
| Unigene5451 | 312 | homeobox protein, putative [Ricinus communis] |
| Unigene4598 | 312 | CDPK-related protein kinase [Gossypium hirsutum] |
| Unigene19708 | 312 | - |
| Unigene16233 | 312 | predicted protein [Populus trichocarpa] |
| Unigene23860 | 312 | - |
| CL229.Contig8 | 242 | alpha tubulin 1 [Pseudotsuga menziesii var. menziesii] |
| Unigene839 | 174 | thioredoxin M-type, chloroplastic [Vitis vinifera] |
| Unigene967 | 153 | predicted protein [Populus trichocarpa] |
| CL3979.Contig1 | 143 | glycine-rich protein precursor [Nicotiana tabacum] |
| CL1704.Contig1 | 125 | peroxidase [Vitis vinifera] |
| Unigene1222 | 115 | Auxin-binding protein ABP20 |
|  | |  |
| **Down regulated** | |  |
| Unigene17381 | 295 | - |
| Unigene10111 | 295 | alpha-soluble NSF attachment protein [Vitis vinifera] |
| Unigene4003 | 295 | protein notum homolog [Glycine max] |
| Unigene3494 | 317 | hypothetical protein VITISV_015697 [Vitis vinifera] |
| CL4128.Contig2 | 317 | uncharacterized methyltransferase At2g41040, chloroplastic-like [Glycine max] |
| CL4377.Contig1 | 317 | f-box family protein [Populus trichocarpa] |
| Unigene11742 | 340 | - |
| Unigene5282 | 340 | beta-1,4-mannosyl-glycoprotein 4-beta-N-acetylglucosaminyltransferase [Vitis vinifera] |
| Unigene13025 | 340 | conserved hypothetical protein [Ricinus communis] |
| Unigene12052 | 363 | - |
| Unigene1760 | 363 | glyceraldehyde-3-phosphate dehydrogenase B [Pyrus x bretschneideri] |
| Unigene6946 | 363 | - |
| Unigene21906 | 363 | - |
| CL322.Contig1 | 385 | uncharacterized protein LOC100257088 [Vitis vinifera] |
| Unigene5059 | 385 | - |
| CL2382.Contig1 | 385 | CRAL-TRIO domain-containing protein YKL091C-like [Vitis vinifera] |
| Unigene18178 | 408 | seed maturation protein [Glycine tomentella] |
| Unigene6216 | 431 | uncharacterized protein LOC100264843 [Vitis vinifera] |
| Unigene12342 | 431 | - |
| Unigene3142 | 476 | aminotransferase family protein [Populus trichocarpa] |
| Unigene19209 | 476 | transcription regulator, putative [Ricinus communis] |
| Unigene12938 | 499 | - |
| Unigene9592 | 522 | uncharacterized protein LOC100854700 [Vitis vinifera] |
| Unigene18407 | 567 | - |
| Unigene8511 | 567 | unknown [Arabidopsis thaliana] |
| Unigene6673 | 1043 | - |
| Unigene7900 | 2585 | - |
