# Supplementary material for: Identification of drought stress-responsive transcription factors in ramie (Boehmeria nivea L. Gaud)
Source: BMC Plant Biol. 2013 Sep 10;13:130. doi: 10.1186/1471-2229-13-130 (PMC3846573; doi:10.1186/1471-2229-13-130)
Supplement: Additional file 3 — Potential pathways affected by drought stress. [file 1471-2229-13-130-S3.doc]

| Pathway | Background number | Gene number regulated | Pvalue | Qvalue | Pathway ID |
| --- | --- | --- | --- | --- | --- |
| [Synthesis and degradation of ketone bodies](../../../../D:%5C2012%5C%E8%AE%BA%E6%96%87%E5%8F%91%E8%A1%A8%5CDGE%5C%E6%95%B0%E6%8D%AE%5Cupload%5Cpathway%5C%E6%96%B0%E5%BB%BA%20Microsoft%20Excel%20%E5%B7%A5%E4%BD%9C%E8%A1%A8.xls#RANGE!gene71%23RANGE!gene71) | 18 | 1 | 0.551 | 0.884 | ko00072 |
| [Glycosphingolipid biosynthesis - globo series](../../../../D:%5C2012%5C%E8%AE%BA%E6%96%87%E5%8F%91%E8%A1%A8%5CDGE%5C%E6%95%B0%E6%8D%AE%5Cupload%5Cpathway%5C%E6%96%B0%E5%BB%BA%20Microsoft%20Excel%20%E5%B7%A5%E4%BD%9C%E8%A1%A8.xls#RANGE!gene68%23RANGE!gene68) | 17 | 1 | 0.530 | 0.884 | ko00603 |
| [Indole alkaloid biosynthesis](../../../../D:%5C2012%5C%E8%AE%BA%E6%96%87%E5%8F%91%E8%A1%A8%5CDGE%5C%E6%95%B0%E6%8D%AE%5Cupload%5Cpathway%5C%E6%96%B0%E5%BB%BA%20Microsoft%20Excel%20%E5%B7%A5%E4%BD%9C%E8%A1%A8.xls#RANGE!gene81%23RANGE!gene81) | 24 | 1 | 0.656 | 0.923 | ko00901 |
| [Photosynthesis - antenna proteins](../../../../D:%5C2012%5C%E8%AE%BA%E6%96%87%E5%8F%91%E8%A1%A8%5CDGE%5C%E6%95%B0%E6%8D%AE%5Cupload%5Cpathway%5C%E6%96%B0%E5%BB%BA%20Microsoft%20Excel%20%E5%B7%A5%E4%BD%9C%E8%A1%A8.xls#RANGE!gene92%23RANGE!gene92) | 37 | 1 | 0.807 | 0.995 | ko00196 |
| [Folate biosynthesis](../../../../D:%5C2012%5C%E8%AE%BA%E6%96%87%E5%8F%91%E8%A1%A8%5CDGE%5C%E6%95%B0%E6%8D%AE%5Cupload%5Cpathway%5C%E6%96%B0%E5%BB%BA%20Microsoft%20Excel%20%E5%B7%A5%E4%BD%9C%E8%A1%A8.xls#RANGE!gene87%23RANGE!gene87) | 33 | 1 | 0.769 | 0.995 | ko00790 |
| [Monoterpenoid biosynthesis](../../../../D:%5C2012%5C%E8%AE%BA%E6%96%87%E5%8F%91%E8%A1%A8%5CDGE%5C%E6%95%B0%E6%8D%AE%5Cupload%5Cpathway%5C%E6%96%B0%E5%BB%BA%20Microsoft%20Excel%20%E5%B7%A5%E4%BD%9C%E8%A1%A8.xls#RANGE!gene90%23RANGE!gene90) | 35 | 1 | 0.789 | 0.995 | ko00902 |
| [Inositol phosphate metabolism](../../../../D:%5C2012%5C%E8%AE%BA%E6%96%87%E5%8F%91%E8%A1%A8%5CDGE%5C%E6%95%B0%E6%8D%AE%5Cupload%5Cpathway%5C%E6%96%B0%E5%BB%BA%20Microsoft%20Excel%20%E5%B7%A5%E4%BD%9C%E8%A1%A8.xls#RANGE!gene113%23RANGE!gene113) | 144 | 1 | 0.998 | 1.000 | ko00562 |
| [Butanoate metabolism](../../../../D:%5C2012%5C%E8%AE%BA%E6%96%87%E5%8F%91%E8%A1%A8%5CDGE%5C%E6%95%B0%E6%8D%AE%5Cupload%5Cpathway%5C%E6%96%B0%E5%BB%BA%20Microsoft%20Excel%20%E5%B7%A5%E4%BD%9C%E8%A1%A8.xls#RANGE!gene100%23RANGE!gene100) | 49 | 1 | 0.887 | 1.000 | ko00650 |
| [Proteasome](../../../../D:%5C2012%5C%E8%AE%BA%E6%96%87%E5%8F%91%E8%A1%A8%5CDGE%5C%E6%95%B0%E6%8D%AE%5Cupload%5Cpathway%5C%E6%96%B0%E5%BB%BA%20Microsoft%20Excel%20%E5%B7%A5%E4%BD%9C%E8%A1%A8.xls#RANGE!gene103%23RANGE!gene103) | 56 | 1 | 0.917 | 1.000 | ko03050 |
| [Base excision repair](../../../../D:%5C2012%5C%E8%AE%BA%E6%96%87%E5%8F%91%E8%A1%A8%5CDGE%5C%E6%95%B0%E6%8D%AE%5Cupload%5Cpathway%5C%E6%96%B0%E5%BB%BA%20Microsoft%20Excel%20%E5%B7%A5%E4%BD%9C%E8%A1%A8.xls#RANGE!gene112%23RANGE!gene112) | 113 | 1 | 0.993 | 1.000 | ko03410 |
| [Nucleotide excision repair](../../../../D:%5C2012%5C%E8%AE%BA%E6%96%87%E5%8F%91%E8%A1%A8%5CDGE%5C%E6%95%B0%E6%8D%AE%5Cupload%5Cpathway%5C%E6%96%B0%E5%BB%BA%20Microsoft%20Excel%20%E5%B7%A5%E4%BD%9C%E8%A1%A8.xls#RANGE!gene114%23RANGE!gene114) | 179 | 1 | 1.000 | 1.000 | ko0342 |
| [Mismatch repair](../../../../D:%5C2012%5C%E8%AE%BA%E6%96%87%E5%8F%91%E8%A1%A8%5CDGE%5C%E6%95%B0%E6%8D%AE%5Cupload%5Cpathway%5C%E6%96%B0%E5%BB%BA%20Microsoft%20Excel%20%E5%B7%A5%E4%BD%9C%E8%A1%A8.xls#RANGE!gene110%23RANGE!gene110) | 103 | 1 | 0.990 | 1.000 | ko03430 |
| [Non-homologous end-joining](../../../../D:%5C2012%5C%E8%AE%BA%E6%96%87%E5%8F%91%E8%A1%A8%5CDGE%5C%E6%95%B0%E6%8D%AE%5Cupload%5Cpathway%5C%E6%96%B0%E5%BB%BA%20Microsoft%20Excel%20%E5%B7%A5%E4%BD%9C%E8%A1%A8.xls#RANGE!gene94%23RANGE!gene94) | 41 | 1 | 0.838 | 1.000 | ko03450 |
| [Arachidonic acid metabolism](../../../../D:%5C2012%5C%E8%AE%BA%E6%96%87%E5%8F%91%E8%A1%A8%5CDGE%5C%E6%95%B0%E6%8D%AE%5Cupload%5Cpathway%5C%E6%96%B0%E5%BB%BA%20Microsoft%20Excel%20%E5%B7%A5%E4%BD%9C%E8%A1%A8.xls#RANGE!gene55%23RANGE!gene55) | 30 | 2 | 0.377 | 0.736 | ko00590 |
| [Vitamin B6 metabolism](../../../../D:%5C2012%5C%E8%AE%BA%E6%96%87%E5%8F%91%E8%A1%A8%5CDGE%5C%E6%95%B0%E6%8D%AE%5Cupload%5Cpathway%5C%E6%96%B0%E5%BB%BA%20Microsoft%20Excel%20%E5%B7%A5%E4%BD%9C%E8%A1%A8.xls#RANGE!gene56%23RANGE!gene56) | 30 | 2 | 0.377 | 0.736 | ko00750 |
| [Other types of O-glycan biosynthesis](../../../../D:%5C2012%5C%E8%AE%BA%E6%96%87%E5%8F%91%E8%A1%A8%5CDGE%5C%E6%95%B0%E6%8D%AE%5Cupload%5Cpathway%5C%E6%96%B0%E5%BB%BA%20Microsoft%20Excel%20%E5%B7%A5%E4%BD%9C%E8%A1%A8.xls#RANGE!gene62%23RANGE!gene62) | 32 | 2 | 0.408 | 0.750 | ko00514 |
| [Brassinosteroid biosynthesis](../../../../D:%5C2012%5C%E8%AE%BA%E6%96%87%E5%8F%91%E8%A1%A8%5CDGE%5C%E6%95%B0%E6%8D%AE%5Cupload%5Cpathway%5C%E6%96%B0%E5%BB%BA%20Microsoft%20Excel%20%E5%B7%A5%E4%BD%9C%E8%A1%A8.xls#RANGE!gene74%23RANGE!gene74) | 44 | 2 | 0.575 | 0.886 | ko00905 |
| [Tropane, piperidine and pyridine alkaloid biosynthesis](../../../../D:%5C2012%5C%E8%AE%BA%E6%96%87%E5%8F%91%E8%A1%A8%5CDGE%5C%E6%95%B0%E6%8D%AE%5Cupload%5Cpathway%5C%E6%96%B0%E5%BB%BA%20Microsoft%20Excel%20%E5%B7%A5%E4%BD%9C%E8%A1%A8.xls#RANGE!gene77%23RANGE!gene77) | 48 | 2 | 0.623 | 0.919 | ko00960 |
| [Lysine degradation](../../../../D:%5C2012%5C%E8%AE%BA%E6%96%87%E5%8F%91%E8%A1%A8%5CDGE%5C%E6%95%B0%E6%8D%AE%5Cupload%5Cpathway%5C%E6%96%B0%E5%BB%BA%20Microsoft%20Excel%20%E5%B7%A5%E4%BD%9C%E8%A1%A8.xls#RANGE!gene80%23RANGE!gene80) | 50 | 2 | 0.645 | 0.920 | ko00310 |
| [Isoflavonoid biosynthesis](../../../../D:%5C2012%5C%E8%AE%BA%E6%96%87%E5%8F%91%E8%A1%A8%5CDGE%5C%E6%95%B0%E6%8D%AE%5Cupload%5Cpathway%5C%E6%96%B0%E5%BB%BA%20Microsoft%20Excel%20%E5%B7%A5%E4%BD%9C%E8%A1%A8.xls#RANGE!gene82%23RANGE!gene82) | 55 | 2 | 0.696 | 0.957 | ko00943 |
| [Benzoxazinoid biosynthesis](../../../../D:%5C2012%5C%E8%AE%BA%E6%96%87%E5%8F%91%E8%A1%A8%5CDGE%5C%E6%95%B0%E6%8D%AE%5Cupload%5Cpathway%5C%E6%96%B0%E5%BB%BA%20Microsoft%20Excel%20%E5%B7%A5%E4%BD%9C%E8%A1%A8.xls#RANGE!gene85%23RANGE!gene85) | 60 | 2 | 0.741 | 0.994 | ko00402 |
| [Steroid biosynthesis](../../../../D:%5C2012%5C%E8%AE%BA%E6%96%87%E5%8F%91%E8%A1%A8%5CDGE%5C%E6%95%B0%E6%8D%AE%5Cupload%5Cpathway%5C%E6%96%B0%E5%BB%BA%20Microsoft%20Excel%20%E5%B7%A5%E4%BD%9C%E8%A1%A8.xls#RANGE!gene91%23RANGE!gene91) | 68 | 2 | 0.801 | 0.995 | ko00100 |
| [Linoleic acid metabolism](../../../../D:%5C2012%5C%E8%AE%BA%E6%96%87%E5%8F%91%E8%A1%A8%5CDGE%5C%E6%95%B0%E6%8D%AE%5Cupload%5Cpathway%5C%E6%96%B0%E5%BB%BA%20Microsoft%20Excel%20%E5%B7%A5%E4%BD%9C%E8%A1%A8.xls#RANGE!gene89%23RANGE!gene89) | 66 | 2 | 0.787 | 0.995 | ko00591 |
| [Citrate cycle (TCA cycle)](../../../../D:%5C2012%5C%E8%AE%BA%E6%96%87%E5%8F%91%E8%A1%A8%5CDGE%5C%E6%95%B0%E6%8D%AE%5Cupload%5Cpathway%5C%E6%96%B0%E5%BB%BA%20Microsoft%20Excel%20%E5%B7%A5%E4%BD%9C%E8%A1%A8.xls#RANGE!gene95%23RANGE!gene95) | 80 | 2 | 0.868 | 1.000 | ko00020 |
| [beta-Alanine metabolism](../../../../D:%5C2012%5C%E8%AE%BA%E6%96%87%E5%8F%91%E8%A1%A8%5CDGE%5C%E6%95%B0%E6%8D%AE%5Cupload%5Cpathway%5C%E6%96%B0%E5%BB%BA%20Microsoft%20Excel%20%E5%B7%A5%E4%BD%9C%E8%A1%A8.xls#RANGE!gene101%23RANGE!gene101) | 87 | 2 | 0.897 | 1.000 | ko00410 |
| [Phosphatidylinositol signaling system](../../../../D:%5C2012%5C%E8%AE%BA%E6%96%87%E5%8F%91%E8%A1%A8%5CDGE%5C%E6%95%B0%E6%8D%AE%5Cupload%5Cpathway%5C%E6%96%B0%E5%BB%BA%20Microsoft%20Excel%20%E5%B7%A5%E4%BD%9C%E8%A1%A8.xls#RANGE!gene109%23RANGE!gene109) | 144 | 2 | 0.988 | 1.000 | ko04070 |
| [Selenocompound metabolism](../../../../D:%5C2012%5C%E8%AE%BA%E6%96%87%E5%8F%91%E8%A1%A8%5CDGE%5C%E6%95%B0%E6%8D%AE%5Cupload%5Cpathway%5C%E6%96%B0%E5%BB%BA%20Microsoft%20Excel%20%E5%B7%A5%E4%BD%9C%E8%A1%A8.xls#RANGE!gene27%23RANGE!gene27) | 26 | 3 | 0.101 | 0.415 | ko00450 |
| [One carbon pool by folate](../../../../D:%5C2012%5C%E8%AE%BA%E6%96%87%E5%8F%91%E8%A1%A8%5CDGE%5C%E6%95%B0%E6%8D%AE%5Cupload%5Cpathway%5C%E6%96%B0%E5%BB%BA%20Microsoft%20Excel%20%E5%B7%A5%E4%BD%9C%E8%A1%A8.xls#RANGE!gene36%23RANGE!gene36) | 30 | 3 | 0.140 | 0.443 | ko00670 |
| [Fatty acid elongation in mitochondria](../../../../D:%5C2012%5C%E8%AE%BA%E6%96%87%E5%8F%91%E8%A1%A8%5CDGE%5C%E6%95%B0%E6%8D%AE%5Cupload%5Cpathway%5C%E6%96%B0%E5%BB%BA%20Microsoft%20Excel%20%E5%B7%A5%E4%BD%9C%E8%A1%A8.xls#RANGE!gene54%23RANGE!gene54) | 49 | 3 | 0.359 | 0.736 | ko00062 |
| [Tryptophan metabolism](../../../../D:%5C2012%5C%E8%AE%BA%E6%96%87%E5%8F%91%E8%A1%A8%5CDGE%5C%E6%95%B0%E6%8D%AE%5Cupload%5Cpathway%5C%E6%96%B0%E5%BB%BA%20Microsoft%20Excel%20%E5%B7%A5%E4%BD%9C%E8%A1%A8.xls#RANGE!gene63%23RANGE!gene63) | 56 | 3 | 0.441 | 0.799 | ko00380 |
| [Histidine metabolism](../../../../D:%5C2012%5C%E8%AE%BA%E6%96%87%E5%8F%91%E8%A1%A8%5CDGE%5C%E6%95%B0%E6%8D%AE%5Cupload%5Cpathway%5C%E6%96%B0%E5%BB%BA%20Microsoft%20Excel%20%E5%B7%A5%E4%BD%9C%E8%A1%A8.xls#RANGE!gene66%23RANGE!gene66) | 62 | 3 | 0.509 | 0.879 | ko00340 |
| [Lysine biosynthesis](../../../../D:%5C2012%5C%E8%AE%BA%E6%96%87%E5%8F%91%E8%A1%A8%5CDGE%5C%E6%95%B0%E6%8D%AE%5Cupload%5Cpathway%5C%E6%96%B0%E5%BB%BA%20Microsoft%20Excel%20%E5%B7%A5%E4%BD%9C%E8%A1%A8.xls#RANGE!gene73%23RANGE!gene73) | 68 | 3 | 0.572 | 0.886 | ko00300 |
| [Fatty acid metabolism](../../../../D:%5C2012%5C%E8%AE%BA%E6%96%87%E5%8F%91%E8%A1%A8%5CDGE%5C%E6%95%B0%E6%8D%AE%5Cupload%5Cpathway%5C%E6%96%B0%E5%BB%BA%20Microsoft%20Excel%20%E5%B7%A5%E4%BD%9C%E8%A1%A8.xls#RANGE!gene75%23RANGE!gene75) | 71 | 3 | 0.601 | 0.914 | ko00071 |
| [Sphingolipid metabolism](../../../../D:%5C2012%5C%E8%AE%BA%E6%96%87%E5%8F%91%E8%A1%A8%5CDGE%5C%E6%95%B0%E6%8D%AE%5Cupload%5Cpathway%5C%E6%96%B0%E5%BB%BA%20Microsoft%20Excel%20%E5%B7%A5%E4%BD%9C%E8%A1%A8.xls#RANGE!gene76%23RANGE!gene76) | 73 | 3 | 0.620 | 0.919 | ko00600 |
| [Phenylalanine, tyrosine and tryptophan biosynthesis](../../../../D:%5C2012%5C%E8%AE%BA%E6%96%87%E5%8F%91%E8%A1%A8%5CDGE%5C%E6%95%B0%E6%8D%AE%5Cupload%5Cpathway%5C%E6%96%B0%E5%BB%BA%20Microsoft%20Excel%20%E5%B7%A5%E4%BD%9C%E8%A1%A8.xls#RANGE!gene84%23RANGE!gene84) | 83 | 3 | 0.705 | 0.957 | ko00400 |
| [Propanoate metabolism](../../../../D:%5C2012%5C%E8%AE%BA%E6%96%87%E5%8F%91%E8%A1%A8%5CDGE%5C%E6%95%B0%E6%8D%AE%5Cupload%5Cpathway%5C%E6%96%B0%E5%BB%BA%20Microsoft%20Excel%20%E5%B7%A5%E4%BD%9C%E8%A1%A8.xls#RANGE!gene86%23RANGE!gene86) | 91 | 3 | 0.762 | 0.995 | ko00640 |
| [DNA replication](../../../../D:%5C2012%5C%E8%AE%BA%E6%96%87%E5%8F%91%E8%A1%A8%5CDGE%5C%E6%95%B0%E6%8D%AE%5Cupload%5Cpathway%5C%E6%96%B0%E5%BB%BA%20Microsoft%20Excel%20%E5%B7%A5%E4%BD%9C%E8%A1%A8.xls#RANGE!gene99%23RANGE!gene99) | 116 | 3 | 0.885 | 1.000 | ko03030 |
| [Regulation of autophagy](../../../../D:%5C2012%5C%E8%AE%BA%E6%96%87%E5%8F%91%E8%A1%A8%5CDGE%5C%E6%95%B0%E6%8D%AE%5Cupload%5Cpathway%5C%E6%96%B0%E5%BB%BA%20Microsoft%20Excel%20%E5%B7%A5%E4%BD%9C%E8%A1%A8.xls#RANGE!gene104%23RANGE!gene104) | 134 | 3 | 0.934 | 1.000 | ko04140 |
| [Circadian rhythm - plant](../../../../D:%5C2012%5C%E8%AE%BA%E6%96%87%E5%8F%91%E8%A1%A8%5CDGE%5C%E6%95%B0%E6%8D%AE%5Cupload%5Cpathway%5C%E6%96%B0%E5%BB%BA%20Microsoft%20Excel%20%E5%B7%A5%E4%BD%9C%E8%A1%A8.xls#RANGE!gene106%23RANGE!gene106) | 157 | 3 | 0.969 | 1.000 | ko04712 |
| [Glycosphingolipid biosynthesis - ganglio series](../../../../D:%5C2012%5C%E8%AE%BA%E6%96%87%E5%8F%91%E8%A1%A8%5CDGE%5C%E6%95%B0%E6%8D%AE%5Cupload%5Cpathway%5C%E6%96%B0%E5%BB%BA%20Microsoft%20Excel%20%E5%B7%A5%E4%BD%9C%E8%A1%A8.xls#RANGE!gene26%23RANGE!gene26) | 41 | 4 | 0.101 | 0.415 | ko00604 |
| [Glucosinolate biosynthesis](../../../../D:%5C2012%5C%E8%AE%BA%E6%96%87%E5%8F%91%E8%A1%A8%5CDGE%5C%E6%95%B0%E6%8D%AE%5Cupload%5Cpathway%5C%E6%96%B0%E5%BB%BA%20Microsoft%20Excel%20%E5%B7%A5%E4%BD%9C%E8%A1%A8.xls#RANGE!gene29%23RANGE!gene29) | 43 | 4 | 0.115 | 0.436 | ko00966 |
| [Fatty acid biosynthesis](../../../../D:%5C2012%5C%E8%AE%BA%E6%96%87%E5%8F%91%E8%A1%A8%5CDGE%5C%E6%95%B0%E6%8D%AE%5Cupload%5Cpathway%5C%E6%96%B0%E5%BB%BA%20Microsoft%20Excel%20%E5%B7%A5%E4%BD%9C%E8%A1%A8.xls#RANGE!gene42%23RANGE!gene42) | 52 | 4 | 0.189 | 0.512 | ko00061 |
| [Sulfur metabolism](../../../../D:%5C2012%5C%E8%AE%BA%E6%96%87%E5%8F%91%E8%A1%A8%5CDGE%5C%E6%95%B0%E6%8D%AE%5Cupload%5Cpathway%5C%E6%96%B0%E5%BB%BA%20Microsoft%20Excel%20%E5%B7%A5%E4%BD%9C%E8%A1%A8.xls#RANGE!gene43%23RANGE!gene43) | 53 | 4 | 0.198 | 0.524 | ko00920 |
| [Sesquiterpenoid biosynthesis](../../../../D:%5C2012%5C%E8%AE%BA%E6%96%87%E5%8F%91%E8%A1%A8%5CDGE%5C%E6%95%B0%E6%8D%AE%5Cupload%5Cpathway%5C%E6%96%B0%E5%BB%BA%20Microsoft%20Excel%20%E5%B7%A5%E4%BD%9C%E8%A1%A8.xls#RANGE!gene44%23RANGE!gene44) | 54 | 4 | 0.207 | 0.536 | ko00909 |
| [Valine, leucine and isoleucine biosynthesis](../../../../D:%5C2012%5C%E8%AE%BA%E6%96%87%E5%8F%91%E8%A1%A8%5CDGE%5C%E6%95%B0%E6%8D%AE%5Cupload%5Cpathway%5C%E6%96%B0%E5%BB%BA%20Microsoft%20Excel%20%E5%B7%A5%E4%BD%9C%E8%A1%A8.xls#RANGE!gene46%23RANGE!gene46) | 56 | 4 | 0.225 | 0.558 | ko00290 |
| [Natural killer cell mediated cytotoxicity](../../../../D:%5C2012%5C%E8%AE%BA%E6%96%87%E5%8F%91%E8%A1%A8%5CDGE%5C%E6%95%B0%E6%8D%AE%5Cupload%5Cpathway%5C%E6%96%B0%E5%BB%BA%20Microsoft%20Excel%20%E5%B7%A5%E4%BD%9C%E8%A1%A8.xls#RANGE!gene47%23RANGE!gene47) | 60 | 4 | 0.263 | 0.638 | ko04650 |
| [Pantothenate and CoA biosynthesis](../../../../D:%5C2012%5C%E8%AE%BA%E6%96%87%E5%8F%91%E8%A1%A8%5CDGE%5C%E6%95%B0%E6%8D%AE%5Cupload%5Cpathway%5C%E6%96%B0%E5%BB%BA%20Microsoft%20Excel%20%E5%B7%A5%E4%BD%9C%E8%A1%A8.xls#RANGE!gene59%23RANGE!gene59) | 72 | 4 | 0.382 | 0.736 | ko00770 |
| [Alanine, aspartate and glutamate metabolism](../../../../D:%5C2012%5C%E8%AE%BA%E6%96%87%E5%8F%91%E8%A1%A8%5CDGE%5C%E6%95%B0%E6%8D%AE%5Cupload%5Cpathway%5C%E6%96%B0%E5%BB%BA%20Microsoft%20Excel%20%E5%B7%A5%E4%BD%9C%E8%A1%A8.xls#RANGE!gene67%23RANGE!gene67) | 87 | 4 | 0.526 | 0.884 | ko00250 |
| [Protein export](../../../../D:%5C2012%5C%E8%AE%BA%E6%96%87%E5%8F%91%E8%A1%A8%5CDGE%5C%E6%95%B0%E6%8D%AE%5Cupload%5Cpathway%5C%E6%96%B0%E5%BB%BA%20Microsoft%20Excel%20%E5%B7%A5%E4%BD%9C%E8%A1%A8.xls#RANGE!gene70%23RANGE!gene70) | 89 | 4 | 0.544 | 0.884 | ko03060 |
| [Glycosylphosphatidylinositol(GPI)-anchor biosynthesis](../../../../D:%5C2012%5C%E8%AE%BA%E6%96%87%E5%8F%91%E8%A1%A8%5CDGE%5C%E6%95%B0%E6%8D%AE%5Cupload%5Cpathway%5C%E6%96%B0%E5%BB%BA%20Microsoft%20Excel%20%E5%B7%A5%E4%BD%9C%E8%A1%A8.xls#RANGE!gene79%23RANGE!gene79) | 101 | 4 | 0.644 | 0.920 | ko00563 |
| [Cyanoamino acid metabolism](../../../../D:%5C2012%5C%E8%AE%BA%E6%96%87%E5%8F%91%E8%A1%A8%5CDGE%5C%E6%95%B0%E6%8D%AE%5Cupload%5Cpathway%5C%E6%96%B0%E5%BB%BA%20Microsoft%20Excel%20%E5%B7%A5%E4%BD%9C%E8%A1%A8.xls#RANGE!gene93%23RANGE!gene93) | 128 | 4 | 0.812 | 0.995 | ko00460 |
| [Basal transcription factors](../../../../D:%5C2012%5C%E8%AE%BA%E6%96%87%E5%8F%91%E8%A1%A8%5CDGE%5C%E6%95%B0%E6%8D%AE%5Cupload%5Cpathway%5C%E6%96%B0%E5%BB%BA%20Microsoft%20Excel%20%E5%B7%A5%E4%BD%9C%E8%A1%A8.xls#RANGE!gene102%23RANGE!gene102) | 151 | 4 | 0.898 | 1.000 | ko03022 |
| [Homologous recombination](../../../../D:%5C2012%5C%E8%AE%BA%E6%96%87%E5%8F%91%E8%A1%A8%5CDGE%5C%E6%95%B0%E6%8D%AE%5Cupload%5Cpathway%5C%E6%96%B0%E5%BB%BA%20Microsoft%20Excel%20%E5%B7%A5%E4%BD%9C%E8%A1%A8.xls#RANGE!gene98%23RANGE!gene98) | 144 | 4 | 0.877 | 1.000 | ko03440 |
| [Glycosaminoglycan degradation](../../../../D:%5C2012%5C%E8%AE%BA%E6%96%87%E5%8F%91%E8%A1%A8%5CDGE%5C%E6%95%B0%E6%8D%AE%5Cupload%5Cpathway%5C%E6%96%B0%E5%BB%BA%20Microsoft%20Excel%20%E5%B7%A5%E4%BD%9C%E8%A1%A8.xls#RANGE!gene33%23RANGE!gene33) | 62 | 5 | 0.131 | 0.443 | ko00531 |
| [Glyoxylate and dicarboxylate metabolism](../../../../D:%5C2012%5C%E8%AE%BA%E6%96%87%E5%8F%91%E8%A1%A8%5CDGE%5C%E6%95%B0%E6%8D%AE%5Cupload%5Cpathway%5C%E6%96%B0%E5%BB%BA%20Microsoft%20Excel%20%E5%B7%A5%E4%BD%9C%E8%A1%A8.xls#RANGE!gene53%23RANGE!gene53) | 89 | 5 | 0.344 | 0.736 | ko00630 |
| [Porphyrin and chlorophyll metabolism](../../../../D:%5C2012%5C%E8%AE%BA%E6%96%87%E5%8F%91%E8%A1%A8%5CDGE%5C%E6%95%B0%E6%8D%AE%5Cupload%5Cpathway%5C%E6%96%B0%E5%BB%BA%20Microsoft%20Excel%20%E5%B7%A5%E4%BD%9C%E8%A1%A8.xls#RANGE!gene69%23RANGE!gene69) | 112 | 5 | 0.539 | 0.884 | ko00860 |
| [Zeatin biosynthesis](../../../../D:%5C2012%5C%E8%AE%BA%E6%96%87%E5%8F%91%E8%A1%A8%5CDGE%5C%E6%95%B0%E6%8D%AE%5Cupload%5Cpathway%5C%E6%96%B0%E5%BB%BA%20Microsoft%20Excel%20%E5%B7%A5%E4%BD%9C%E8%A1%A8.xls#RANGE!gene97%23RANGE!gene97) | 173 | 5 | 0.876 | 1.000 | ko00908 |
| [Biosynthesis of unsaturated fatty acids](../../../../D:%5C2012%5C%E8%AE%BA%E6%96%87%E5%8F%91%E8%A1%A8%5CDGE%5C%E6%95%B0%E6%8D%AE%5Cupload%5Cpathway%5C%E6%96%B0%E5%BB%BA%20Microsoft%20Excel%20%E5%B7%A5%E4%BD%9C%E8%A1%A8.xls#RANGE!gene20%23RANGE!gene20) | 57 | 6 | 0.037 | 0.200 | ko01040 |
| [N-Glycan biosynthesis](../../../../D:%5C2012%5C%E8%AE%BA%E6%96%87%E5%8F%91%E8%A1%A8%5CDGE%5C%E6%95%B0%E6%8D%AE%5Cupload%5Cpathway%5C%E6%96%B0%E5%BB%BA%20Microsoft%20Excel%20%E5%B7%A5%E4%BD%9C%E8%A1%A8.xls#RANGE!gene28%23RANGE!gene28) | 74 | 6 | 0.102 | 0.415 | ko00510 |
| [Nitrogen metabolism](../../../../D:%5C2012%5C%E8%AE%BA%E6%96%87%E5%8F%91%E8%A1%A8%5CDGE%5C%E6%95%B0%E6%8D%AE%5Cupload%5Cpathway%5C%E6%96%B0%E5%BB%BA%20Microsoft%20Excel%20%E5%B7%A5%E4%BD%9C%E8%A1%A8.xls#RANGE!gene32%23RANGE!gene32) | 78 | 6 | 0.123 | 0.438 | ko00910 |
| [Flavone and flavonol biosynthesis](../../../../D:%5C2012%5C%E8%AE%BA%E6%96%87%E5%8F%91%E8%A1%A8%5CDGE%5C%E6%95%B0%E6%8D%AE%5Cupload%5Cpathway%5C%E6%96%B0%E5%BB%BA%20Microsoft%20Excel%20%E5%B7%A5%E4%BD%9C%E8%A1%A8.xls#RANGE!gene50%23RANGE!gene50) | 108 | 6 | 0.329 | 0.734 | ko00944 |
| [Aminoacyl-tRNA biosynthesis](../../../../D:%5C2012%5C%E8%AE%BA%E6%96%87%E5%8F%91%E8%A1%A8%5CDGE%5C%E6%95%B0%E6%8D%AE%5Cupload%5Cpathway%5C%E6%96%B0%E5%BB%BA%20Microsoft%20Excel%20%E5%B7%A5%E4%BD%9C%E8%A1%A8.xls#RANGE!gene51%23RANGE!gene51) | 108 | 6 | 0.329 | 0.734 | ko00970 |
| [alpha-Linolenic acid metabolism](../../../../D:%5C2012%5C%E8%AE%BA%E6%96%87%E5%8F%91%E8%A1%A8%5CDGE%5C%E6%95%B0%E6%8D%AE%5Cupload%5Cpathway%5C%E6%96%B0%E5%BB%BA%20Microsoft%20Excel%20%E5%B7%A5%E4%BD%9C%E8%A1%A8.xls#RANGE!gene52%23RANGE!gene52) | 110 | 6 | 0.344 | 0.736 | ko00592 |
| [Glutathione metabolism](../../../../D:%5C2012%5C%E8%AE%BA%E6%96%87%E5%8F%91%E8%A1%A8%5CDGE%5C%E6%95%B0%E6%8D%AE%5Cupload%5Cpathway%5C%E6%96%B0%E5%BB%BA%20Microsoft%20Excel%20%E5%B7%A5%E4%BD%9C%E8%A1%A8.xls#RANGE!gene65%23RANGE!gene65) | 130 | 6 | 0.499 | 0.875 | ko00480 |
| [Terpenoid backbone biosynthesis](../../../../D:%5C2012%5C%E8%AE%BA%E6%96%87%E5%8F%91%E8%A1%A8%5CDGE%5C%E6%95%B0%E6%8D%AE%5Cupload%5Cpathway%5C%E6%96%B0%E5%BB%BA%20Microsoft%20Excel%20%E5%B7%A5%E4%BD%9C%E8%A1%A8.xls#RANGE!gene72%23RANGE!gene72) | 139 | 6 | 0.565 | 0.886 | ko00900 |
| [Peroxisome](../../../../D:%5C2012%5C%E8%AE%BA%E6%96%87%E5%8F%91%E8%A1%A8%5CDGE%5C%E6%95%B0%E6%8D%AE%5Cupload%5Cpathway%5C%E6%96%B0%E5%BB%BA%20Microsoft%20Excel%20%E5%B7%A5%E4%BD%9C%E8%A1%A8.xls#RANGE!gene88%23RANGE!gene88) | 174 | 6 | 0.772 | 0.995 | ko04146 |
| [Riboflavin metabolism](../../../../D:%5C2012%5C%E8%AE%BA%E6%96%87%E5%8F%91%E8%A1%A8%5CDGE%5C%E6%95%B0%E6%8D%AE%5Cupload%5Cpathway%5C%E6%96%B0%E5%BB%BA%20Microsoft%20Excel%20%E5%B7%A5%E4%BD%9C%E8%A1%A8.xls#RANGE!gene10%23RANGE!gene10) | 48 | 7 | 0.004 | 0.049 | ko00740 |
| [Isoquinoline alkaloid biosynthesis](../../../../D:%5C2012%5C%E8%AE%BA%E6%96%87%E5%8F%91%E8%A1%A8%5CDGE%5C%E6%95%B0%E6%8D%AE%5Cupload%5Cpathway%5C%E6%96%B0%E5%BB%BA%20Microsoft%20Excel%20%E5%B7%A5%E4%BD%9C%E8%A1%A8.xls#RANGE!gene22%23RANGE!gene22) | 77 | 7 | 0.050 | 0.257 | ko00950 |
| [Ubiquinone and other terpenoid-quinone biosynthesis](../../../../D:%5C2012%5C%E8%AE%BA%E6%96%87%E5%8F%91%E8%A1%A8%5CDGE%5C%E6%95%B0%E6%8D%AE%5Cupload%5Cpathway%5C%E6%96%B0%E5%BB%BA%20Microsoft%20Excel%20%E5%B7%A5%E4%BD%9C%E8%A1%A8.xls#RANGE!gene34%23RANGE!gene34) | 98 | 7 | 0.134 | 0.443 | ko00130 |
| [Tyrosine metabolism](../../../../D:%5C2012%5C%E8%AE%BA%E6%96%87%E5%8F%91%E8%A1%A8%5CDGE%5C%E6%95%B0%E6%8D%AE%5Cupload%5Cpathway%5C%E6%96%B0%E5%BB%BA%20Microsoft%20Excel%20%E5%B7%A5%E4%BD%9C%E8%A1%A8.xls#RANGE!gene57%23RANGE!gene57) | 136 | 7 | 0.378 | 0.736 | ko00350 |
| [Limonene and pinene degradation](../../../../D:%5C2012%5C%E8%AE%BA%E6%96%87%E5%8F%91%E8%A1%A8%5CDGE%5C%E6%95%B0%E6%8D%AE%5Cupload%5Cpathway%5C%E6%96%B0%E5%BB%BA%20Microsoft%20Excel%20%E5%B7%A5%E4%BD%9C%E8%A1%A8.xls#RANGE!gene61%23RANGE!gene61) | 139 | 7 | 0.400 | 0.747 | ko00903 |
| [RNA degradation](../../../../D:%5C2012%5C%E8%AE%BA%E6%96%87%E5%8F%91%E8%A1%A8%5CDGE%5C%E6%95%B0%E6%8D%AE%5Cupload%5Cpathway%5C%E6%96%B0%E5%BB%BA%20Microsoft%20Excel%20%E5%B7%A5%E4%BD%9C%E8%A1%A8.xls#RANGE!gene111%23RANGE!gene111) | 341 | 7 | 0.993 | 1.000 | ko03018 |
| [Glycerolipid metabolism](../../../../D:%5C2012%5C%E8%AE%BA%E6%96%87%E5%8F%91%E8%A1%A8%5CDGE%5C%E6%95%B0%E6%8D%AE%5Cupload%5Cpathway%5C%E6%96%B0%E5%BB%BA%20Microsoft%20Excel%20%E5%B7%A5%E4%BD%9C%E8%A1%A8.xls#RANGE!gene24%23RANGE!gene24) | 102 | 8 | 0.076 | 0.361 | ko00561 |
| [Valine, leucine and isoleucine degradation](../../../../D:%5C2012%5C%E8%AE%BA%E6%96%87%E5%8F%91%E8%A1%A8%5CDGE%5C%E6%95%B0%E6%8D%AE%5Cupload%5Cpathway%5C%E6%96%B0%E5%BB%BA%20Microsoft%20Excel%20%E5%B7%A5%E4%BD%9C%E8%A1%A8.xls#RANGE!gene31%23RANGE!gene31) | 113 | 8 | 0.119 | 0.436 | ko00280 |
| [Glycine, serine and threonine metabolism](../../../../D:%5C2012%5C%E8%AE%BA%E6%96%87%E5%8F%91%E8%A1%A8%5CDGE%5C%E6%95%B0%E6%8D%AE%5Cupload%5Cpathway%5C%E6%96%B0%E5%BB%BA%20Microsoft%20Excel%20%E5%B7%A5%E4%BD%9C%E8%A1%A8.xls#RANGE!gene41%23RANGE!gene41) | 127 | 8 | 0.188 | 0.512 | ko00260 |
| [ABC transporters](../../../../D:%5C2012%5C%E8%AE%BA%E6%96%87%E5%8F%91%E8%A1%A8%5CDGE%5C%E6%95%B0%E6%8D%AE%5Cupload%5Cpathway%5C%E6%96%B0%E5%BB%BA%20Microsoft%20Excel%20%E5%B7%A5%E4%BD%9C%E8%A1%A8.xls#RANGE!gene83%23RANGE!gene83) | 211 | 8 | 0.702 | 0.957 | ko02010 |
| [Oxidative phosphorylation](../../../../D:%5C2012%5C%E8%AE%BA%E6%96%87%E5%8F%91%E8%A1%A8%5CDGE%5C%E6%95%B0%E6%8D%AE%5Cupload%5Cpathway%5C%E6%96%B0%E5%BB%BA%20Microsoft%20Excel%20%E5%B7%A5%E4%BD%9C%E8%A1%A8.xls#RANGE!gene96%23RANGE!gene96) | 256 | 8 | 0.872 | 1.000 | ko00190 |
| [Galactose metabolism](../../../../D:%5C2012%5C%E8%AE%BA%E6%96%87%E5%8F%91%E8%A1%A8%5CDGE%5C%E6%95%B0%E6%8D%AE%5Cupload%5Cpathway%5C%E6%96%B0%E5%BB%BA%20Microsoft%20Excel%20%E5%B7%A5%E4%BD%9C%E8%A1%A8.xls#RANGE!gene37%23RANGE!gene37) | 139 | 9 | 0.151 | 0.466 | ko00052 |
| [Arginine and proline metabolism](../../../../D:%5C2012%5C%E8%AE%BA%E6%96%87%E5%8F%91%E8%A1%A8%5CDGE%5C%E6%95%B0%E6%8D%AE%5Cupload%5Cpathway%5C%E6%96%B0%E5%BB%BA%20Microsoft%20Excel%20%E5%B7%A5%E4%BD%9C%E8%A1%A8.xls#RANGE!gene38%23RANGE!gene38) | 142 | 9 | 0.165 | 0.487 | ko00330 |
| [Cysteine and methionine metabolism](../../../../D:%5C2012%5C%E8%AE%BA%E6%96%87%E5%8F%91%E8%A1%A8%5CDGE%5C%E6%95%B0%E6%8D%AE%5Cupload%5Cpathway%5C%E6%96%B0%E5%BB%BA%20Microsoft%20Excel%20%E5%B7%A5%E4%BD%9C%E8%A1%A8.xls#RANGE!gene60%23RANGE!gene60) | 181 | 9 | 0.388 | 0.736 | ko00270 |
| [Stilbenoid, diarylheptanoid and gingerol biosynthesis](../../../../D:%5C2012%5C%E8%AE%BA%E6%96%87%E5%8F%91%E8%A1%A8%5CDGE%5C%E6%95%B0%E6%8D%AE%5Cupload%5Cpathway%5C%E6%96%B0%E5%BB%BA%20Microsoft%20Excel%20%E5%B7%A5%E4%BD%9C%E8%A1%A8.xls#RANGE!gene58%23RANGE!gene58) | 180 | 9 | 0.381 | 0.736 | ko00945 |
| [Ribosome biogenesis in eukaryotes](../../../../D:%5C2012%5C%E8%AE%BA%E6%96%87%E5%8F%91%E8%A1%A8%5CDGE%5C%E6%95%B0%E6%8D%AE%5Cupload%5Cpathway%5C%E6%96%B0%E5%BB%BA%20Microsoft%20Excel%20%E5%B7%A5%E4%BD%9C%E8%A1%A8.xls#RANGE!gene107%23RANGE!gene107) | 362 | 9 | 0.978 | 1.000 | ko03008 |
| [Pentose phosphate pathway](../../../../D:%5C2012%5C%E8%AE%BA%E6%96%87%E5%8F%91%E8%A1%A8%5CDGE%5C%E6%95%B0%E6%8D%AE%5Cupload%5Cpathway%5C%E6%96%B0%E5%BB%BA%20Microsoft%20Excel%20%E5%B7%A5%E4%BD%9C%E8%A1%A8.xls#RANGE!gene17%23RANGE!gene17) | 117 | 10 | 0.031 | 0.200 | ko00030 |
| [Ubiquitin mediated proteolysis](../../../../D:%5C2012%5C%E8%AE%BA%E6%96%87%E5%8F%91%E8%A1%A8%5CDGE%5C%E6%95%B0%E6%8D%AE%5Cupload%5Cpathway%5C%E6%96%B0%E5%BB%BA%20Microsoft%20Excel%20%E5%B7%A5%E4%BD%9C%E8%A1%A8.xls#RANGE!gene105%23RANGE!gene105) | 348 | 10 | 0.940 | 1.000 | ko04120 |
| [Ascorbate and aldarate metabolism](../../../../D:%5C2012%5C%E8%AE%BA%E6%96%87%E5%8F%91%E8%A1%A8%5CDGE%5C%E6%95%B0%E6%8D%AE%5Cupload%5Cpathway%5C%E6%96%B0%E5%BB%BA%20Microsoft%20Excel%20%E5%B7%A5%E4%BD%9C%E8%A1%A8.xls#RANGE!gene11%23RANGE!gene11) | 102 | 11 | 0.005 | 0.049 | ko00053 |
| [Photosynthesis](../../../../D:%5C2012%5C%E8%AE%BA%E6%96%87%E5%8F%91%E8%A1%A8%5CDGE%5C%E6%95%B0%E6%8D%AE%5Cupload%5Cpathway%5C%E6%96%B0%E5%BB%BA%20Microsoft%20Excel%20%E5%B7%A5%E4%BD%9C%E8%A1%A8.xls#RANGE!gene23%23RANGE!gene23) | 146 | 11 | 0.054 | 0.266 | ko00195 |
| [Flavonoid biosynthesis](../../../../D:%5C2012%5C%E8%AE%BA%E6%96%87%E5%8F%91%E8%A1%A8%5CDGE%5C%E6%95%B0%E6%8D%AE%5Cupload%5Cpathway%5C%E6%96%B0%E5%BB%BA%20Microsoft%20Excel%20%E5%B7%A5%E4%BD%9C%E8%A1%A8.xls#RANGE!gene48%23RANGE!gene48) | 212 | 11 | 0.316 | 0.734 | ko00941 |
| [Phenylalanine metabolism](../../../../D:%5C2012%5C%E8%AE%BA%E6%96%87%E5%8F%91%E8%A1%A8%5CDGE%5C%E6%95%B0%E6%8D%AE%5Cupload%5Cpathway%5C%E6%96%B0%E5%BB%BA%20Microsoft%20Excel%20%E5%B7%A5%E4%BD%9C%E8%A1%A8.xls#RANGE!gene16%23RANGE!gene16) | 150 | 12 | 0.031 | 0.200 | ko00360 |
| [Pyruvate metabolism](../../../../D:%5C2012%5C%E8%AE%BA%E6%96%87%E5%8F%91%E8%A1%A8%5CDGE%5C%E6%95%B0%E6%8D%AE%5Cupload%5Cpathway%5C%E6%96%B0%E5%BB%BA%20Microsoft%20Excel%20%E5%B7%A5%E4%BD%9C%E8%A1%A8.xls#RANGE!gene15%23RANGE!gene15) | 160 | 13 | 0.022 | 0.170 | ko00620 |
| [Fructose and mannose metabolism](../../../../D:%5C2012%5C%E8%AE%BA%E6%96%87%E5%8F%91%E8%A1%A8%5CDGE%5C%E6%95%B0%E6%8D%AE%5Cupload%5Cpathway%5C%E6%96%B0%E5%BB%BA%20Microsoft%20Excel%20%E5%B7%A5%E4%BD%9C%E8%A1%A8.xls#RANGE!gene7%23RANGE!gene7) | 136 | 14 | 0.002 | 0.039 | ko00051 |
| [RNA polymerase](../../../../D:%5C2012%5C%E8%AE%BA%E6%96%87%E5%8F%91%E8%A1%A8%5CDGE%5C%E6%95%B0%E6%8D%AE%5Cupload%5Cpathway%5C%E6%96%B0%E5%BB%BA%20Microsoft%20Excel%20%E5%B7%A5%E4%BD%9C%E8%A1%A8.xls#RANGE!gene35%23RANGE!gene35) | 233 | 14 | 0.138 | 0.443 | ko03020 |
| [Carotenoid biosynthesis](../../../../D:%5C2012%5C%E8%AE%BA%E6%96%87%E5%8F%91%E8%A1%A8%5CDGE%5C%E6%95%B0%E6%8D%AE%5Cupload%5Cpathway%5C%E6%96%B0%E5%BB%BA%20Microsoft%20Excel%20%E5%B7%A5%E4%BD%9C%E8%A1%A8.xls#RANGE!gene12%23RANGE!gene12) | 165 | 15 | 0.006 | 0.053 | ko00906 |
| [Glycolysis / Gluconeogenesis](../../../../D:%5C2012%5C%E8%AE%BA%E6%96%87%E5%8F%91%E8%A1%A8%5CDGE%5C%E6%95%B0%E6%8D%AE%5Cupload%5Cpathway%5C%E6%96%B0%E5%BB%BA%20Microsoft%20Excel%20%E5%B7%A5%E4%BD%9C%E8%A1%A8.xls#RANGE!gene21%23RANGE!gene21) | 225 | 16 | 0.037 | 0.200 | ko00010 |
| [Carbon fixation in photosynthetic organisms](../../../../D:%5C2012%5C%E8%AE%BA%E6%96%87%E5%8F%91%E8%A1%A8%5CDGE%5C%E6%95%B0%E6%8D%AE%5Cupload%5Cpathway%5C%E6%96%B0%E5%BB%BA%20Microsoft%20Excel%20%E5%B7%A5%E4%BD%9C%E8%A1%A8.xls#RANGE!gene4%23RANGE!gene4) | 144 | 17 | 0.000 | 0.005 | ko00710 |
| [Amino sugar and nucleotide sugar metabolism](../../../../D:%5C2012%5C%E8%AE%BA%E6%96%87%E5%8F%91%E8%A1%A8%5CDGE%5C%E6%95%B0%E6%8D%AE%5Cupload%5Cpathway%5C%E6%96%B0%E5%BB%BA%20Microsoft%20Excel%20%E5%B7%A5%E4%BD%9C%E8%A1%A8.xls#RANGE!gene13%23RANGE!gene13) | 219 | 18 | 0.007 | 0.064 | ko00520 |
| [Other glycan degradation](../../../../D:%5C2012%5C%E8%AE%BA%E6%96%87%E5%8F%91%E8%A1%A8%5CDGE%5C%E6%95%B0%E6%8D%AE%5Cupload%5Cpathway%5C%E6%96%B0%E5%BB%BA%20Microsoft%20Excel%20%E5%B7%A5%E4%BD%9C%E8%A1%A8.xls#RANGE!gene3%23RANGE!gene3) | 123 | 19 | 0.000 | 0.000 | ko00511 |
| [Phenylpropanoid biosynthesis](../../../../D:%5C2012%5C%E8%AE%BA%E6%96%87%E5%8F%91%E8%A1%A8%5CDGE%5C%E6%95%B0%E6%8D%AE%5Cupload%5Cpathway%5C%E6%96%B0%E5%BB%BA%20Microsoft%20Excel%20%E5%B7%A5%E4%BD%9C%E8%A1%A8.xls#RANGE!gene39%23RANGE!gene39) | 343 | 19 | 0.167 | 0.487 | ko00940 |
| [Pyrimidine metabolism](../../../../D:%5C2012%5C%E8%AE%BA%E6%96%87%E5%8F%91%E8%A1%A8%5CDGE%5C%E6%95%B0%E6%8D%AE%5Cupload%5Cpathway%5C%E6%96%B0%E5%BB%BA%20Microsoft%20Excel%20%E5%B7%A5%E4%BD%9C%E8%A1%A8.xls#RANGE!gene49%23RANGE!gene49) | 387 | 19 | 0.324 | 0.734 | ko00240 |
| [Phagosome](../../../../D:%5C2012%5C%E8%AE%BA%E6%96%87%E5%8F%91%E8%A1%A8%5CDGE%5C%E6%95%B0%E6%8D%AE%5Cupload%5Cpathway%5C%E6%96%B0%E5%BB%BA%20Microsoft%20Excel%20%E5%B7%A5%E4%BD%9C%E8%A1%A8.xls#RANGE!gene5%23RANGE!gene5) | 238 | 22 | 0.001 | 0.017 | ko04145 |
| [Spliceosome](../../../../D:%5C2012%5C%E8%AE%BA%E6%96%87%E5%8F%91%E8%A1%A8%5CDGE%5C%E6%95%B0%E6%8D%AE%5Cupload%5Cpathway%5C%E6%96%B0%E5%BB%BA%20Microsoft%20Excel%20%E5%B7%A5%E4%BD%9C%E8%A1%A8.xls#RANGE!gene108%23RANGE!gene108) | 745 | 22 | 0.981 | 1.000 | ko03040 |
| [Purine metabolism](../../../../D:%5C2012%5C%E8%AE%BA%E6%96%87%E5%8F%91%E8%A1%A8%5CDGE%5C%E6%95%B0%E6%8D%AE%5Cupload%5Cpathway%5C%E6%96%B0%E5%BB%BA%20Microsoft%20Excel%20%E5%B7%A5%E4%BD%9C%E8%A1%A8.xls#RANGE!gene30%23RANGE!gene30) | 426 | 24 | 0.117 | 0.436 | ko00230 |
| [Pentose and glucuronate interconversions](../../../../D:%5C2012%5C%E8%AE%BA%E6%96%87%E5%8F%91%E8%A1%A8%5CDGE%5C%E6%95%B0%E6%8D%AE%5Cupload%5Cpathway%5C%E6%96%B0%E5%BB%BA%20Microsoft%20Excel%20%E5%B7%A5%E4%BD%9C%E8%A1%A8.xls#RANGE!gene6%23RANGE!gene6) | 320 | 26 | 0.002 | 0.032 | ko00040 |
| [Protein processing in endoplasmic reticulum](../../../../D:%5C2012%5C%E8%AE%BA%E6%96%87%E5%8F%91%E8%A1%A8%5CDGE%5C%E6%95%B0%E6%8D%AE%5Cupload%5Cpathway%5C%E6%96%B0%E5%BB%BA%20Microsoft%20Excel%20%E5%B7%A5%E4%BD%9C%E8%A1%A8.xls#RANGE!gene40%23RANGE!gene40) | 493 | 26 | 0.179 | 0.509 | ko04141 |
| [Starch and sucrose metabolism](../../../../D:%5C2012%5C%E8%AE%BA%E6%96%87%E5%8F%91%E8%A1%A8%5CDGE%5C%E6%95%B0%E6%8D%AE%5Cupload%5Cpathway%5C%E6%96%B0%E5%BB%BA%20Microsoft%20Excel%20%E5%B7%A5%E4%BD%9C%E8%A1%A8.xls#RANGE!gene9%23RANGE!gene9) | 565 | 39 | 0.003 | 0.039 | ko00500 |
| [mRNA surveillance pathway](../../../../D:%5C2012%5C%E8%AE%BA%E6%96%87%E5%8F%91%E8%A1%A8%5CDGE%5C%E6%95%B0%E6%8D%AE%5Cupload%5Cpathway%5C%E6%96%B0%E5%BB%BA%20Microsoft%20Excel%20%E5%B7%A5%E4%BD%9C%E8%A1%A8.xls#RANGE!gene64%23RANGE!gene64) | 942 | 42 | 0.453 | 0.807 | ko03015 |
| [Ether lipid metabolism](../../../../D:%5C2012%5C%E8%AE%BA%E6%96%87%E5%8F%91%E8%A1%A8%5CDGE%5C%E6%95%B0%E6%8D%AE%5Cupload%5Cpathway%5C%E6%96%B0%E5%BB%BA%20Microsoft%20Excel%20%E5%B7%A5%E4%BD%9C%E8%A1%A8.xls#RANGE!gene18%23RANGE!gene18) | 799 | 46 | 0.032 | 0.200 | ko00565 |
| [Plant-pathogen interaction](../../../../D:%5C2012%5C%E8%AE%BA%E6%96%87%E5%8F%91%E8%A1%A8%5CDGE%5C%E6%95%B0%E6%8D%AE%5Cupload%5Cpathway%5C%E6%96%B0%E5%BB%BA%20Microsoft%20Excel%20%E5%B7%A5%E4%BD%9C%E8%A1%A8.xls#RANGE!gene78%23RANGE!gene78) | 1147 | 48 | 0.629 | 0.919 | ko04626 |
| [Plant hormone signal transduction](../../../../D:%5C2012%5C%E8%AE%BA%E6%96%87%E5%8F%91%E8%A1%A8%5CDGE%5C%E6%95%B0%E6%8D%AE%5Cupload%5Cpathway%5C%E6%96%B0%E5%BB%BA%20Microsoft%20Excel%20%E5%B7%A5%E4%BD%9C%E8%A1%A8.xls#RANGE!gene25%23RANGE!gene25) | 928 | 49 | 0.091 | 0.415 | ko04075 |
| [Glycerophospholipid metabolism](../../../../D:%5C2012%5C%E8%AE%BA%E6%96%87%E5%8F%91%E8%A1%A8%5CDGE%5C%E6%95%B0%E6%8D%AE%5Cupload%5Cpathway%5C%E6%96%B0%E5%BB%BA%20Microsoft%20Excel%20%E5%B7%A5%E4%BD%9C%E8%A1%A8.xls#RANGE!gene19%23RANGE!gene19) | 947 | 53 | 0.035 | 0.200 | ko00564 |
| [Ribosome](../../../../D:%5C2012%5C%E8%AE%BA%E6%96%87%E5%8F%91%E8%A1%A8%5CDGE%5C%E6%95%B0%E6%8D%AE%5Cupload%5Cpathway%5C%E6%96%B0%E5%BB%BA%20Microsoft%20Excel%20%E5%B7%A5%E4%BD%9C%E8%A1%A8.xls#RANGE!gene1%23RANGE!gene1) | 354 | 55 | 0.000 | 0.000 | ko03010 |
| [Endocytosis](../../../../D:%5C2012%5C%E8%AE%BA%E6%96%87%E5%8F%91%E8%A1%A8%5CDGE%5C%E6%95%B0%E6%8D%AE%5Cupload%5Cpathway%5C%E6%96%B0%E5%BB%BA%20Microsoft%20Excel%20%E5%B7%A5%E4%BD%9C%E8%A1%A8.xls#RANGE!gene14%23RANGE!gene14) | 979 | 57 | 0.015 | 0.122 | ko04144 |
| [RNA transport](../../../../D:%5C2012%5C%E8%AE%BA%E6%96%87%E5%8F%91%E8%A1%A8%5CDGE%5C%E6%95%B0%E6%8D%AE%5Cupload%5Cpathway%5C%E6%96%B0%E5%BB%BA%20Microsoft%20Excel%20%E5%B7%A5%E4%BD%9C%E8%A1%A8.xls#RANGE!gene45%23RANGE!gene45) | 1287 | 62 | 0.212 | 0.537 | ko03013 |
