# Supplementary material for: Identification of drought stress-responsive transcription factors in ramie (Boehmeria nivea L. Gaud)
Source: BMC Plant Biol. 2013 Sep 10;13:130. doi: 10.1186/1471-2229-13-130 (PMC3846573; doi:10.1186/1471-2229-13-130)
Supplement: Additional file 4 — Potential drought stress-responsive transcription factors. [file 1471-2229-13-130-S4.doc]

| Gene | Fold | Annotation by blast Nr database |
| --- | --- | --- |
| **Up-regulated** | |  |
| Unigene4099 | 2.1 | transcription factor UNE10-like [Glycine max] |
| Unigene2486 | 2.2 | GATA transcription factor, putative [Ricinus communis] |
| CL2692.Contig1 | 2.3 | domain class transcription factor [Malus x domestica] |
| Unigene957 | 2.4 | AP2 domain class transcription factor [Malus x domestica] |
| Unigene7751 | 2.4 | zinc finger protein, putative [Ricinus communis] |
| Unigene11226 | 3.3 | transcription factor TCP19-like [Glycine max] |
| Unigene8373 | 3.6 | NAC domain-containing protein 100-like [Vitis vinifera] |
| Unigene19206 | 4.0 | hypothetical transcription factor [Prunus persica] |
| Unigene13048 | 4.4 | homeobox-leucine zipper protein HAT14-like [Vitis vinifera] |
| CL3391.Contig1 | 5.1 | myb-related protein 306-like [Glycine max] |
| Unigene2022 | 5.3 | C2H2L domain class transcription factor [Malus x domestica] |
| CL5149.Contig1 | 6.9 | homeobox protein, putative [Ricinus communis] |
| Unigene8530 | 7.7 | DOF domain class transcription factor [Malus x domestica] |
| Unigene7003 | 8.3 | transcription factor bHLH87-like [Vitis vinifera] |
| Unigene13775 | 10.4 | NAC domain-containing protein 7-like [Glycine max] |
| Unigene4573 | 37.2 | ethylene responsive transcription factor 1a [Prunus salicina] |
| Unigene5451 | 312 | homeobox protein, putative [Ricinus communis] |
| Unigene19721 | 335 | DELLA protein RGL1, putative [Ricinus communis] |
| Unigene9044 | 335 | NAC domain-containing protein 8 [Vitis vinifera] |
| Unigene2547 | 357 | homeobox protein knotted-1-like 7-like [Glycine max] |
| **Down-regulated** | |  |
| Unigene565 | 4.2 | homeobox-leucine zipper protein ATHB-16-like, partial [Glycine max] |
| Unigene1569 | 4.9 | myb transcription factor [Humulus lupulus] |
| Unigene5955 | 8.6 | auxin-responsive family protein [Arabidopsis lyrata subsp. lyrata] |
| Unigene19209 | 476 | transcription regulator, putative [Ricinus communis] |
