# Supplementary material for: Identification of drought stress-responsive transcription factors in ramie (Boehmeria nivea L. Gaud)
Source: BMC Plant Biol. 2013 Sep 10;13:130. doi: 10.1186/1471-2229-13-130 (PMC3846573; doi:10.1186/1471-2229-13-130)
Supplement: Additional file 5 — Primers of genes validated by qRT-PCR. [file 1471-2229-13-130-S5.doc]

| Gene | Forward primer (5'-3') | Reverse primer (5'-3') |
| --- | --- | --- |
| Unigene1369 | CCTCCAACAACGGTTCTCAT | ACCCAAATGGCAAAGAACAG |
| Unigene4099 | CAAAGAATGAGGACAGGGGA | ACGTAGACGACGTGTGTTGG |
| Unigene8530 | TCAGGAGTGGGTTCTTGGAG | GATTGTGGTCACCGTCACTG |
| Unigene2022 | GACATGTGGTTAACCGAGGC | TAACAACATCTTCTCCGGGC |
| Unigene957 | GCCCTTAGGTTCGATCACC | TGAGATCGAGGTCGAGGACT |
| Unigene1078 | GTAGCTTGACCGGTGCTCAT | GTTGGGGATCCTGTGAGAGA |
| Unigene9044 | CAAGCAAGGGAAAGCTTGAG | AACCACATGAAGCAGCAACA |
| Unigene13775 | AGTTGAGCCACGATCAGGAC | ATAAACGACGCCGTATCCAA |
| Unigene8373 | ACAATGTTCCGTTTCCGTTC | CGACTGGTCTTGCATCAGAA |
| Unigene19721 | CCTCAGGTCTTCCACGTCTC | CCTCGAAAGCTCTCAGCAAC |
| Unigene10248 | TTGCTCATTGTTGCAAGGAC | AGGGCACAACGGCATAGTAG |
| Unigene565 | TCTCTCGGCGAAGGAGATTA | GTACGACGACGAAGACGACA |
| Unigene1569 | AGAGAAATGGGGGTGCTTTT | CTCCTTCTGCTCCTCCCTCT |
| Unigene5955 | AGCTAAGAGGCAAGCTCGTG | ATGCTCCAGTGGGACGTAAC |
| Unigene5390 | CATCGGCACCATTGTTATTG | TGTCACGGTACTTCCCCTTC |
| Unigene19209 | AAGTGGGCATGGATTTCTTG | AACACTTGGGGGCTTTTTCT |
| CL4185.Contig1 | AAAGTGACGTTGGGGAACTG | GACAAGGCCAAAATCAAGGA |
| CL4538.Contig1 | GAGTTCGGAGAGCTGGAATG | CTGTGTGAGGCAGCATTGTT |
| CL4377.Contig1 | TTCAGGCAAAGGAATCCATC | TGGACAAAGGGAGTGGAAAG |
| Unigene738 | CGTCATTCTTTGTGGTGGTG | GACGAAGAGAAAAAGCACCG |
| Actin | TAACATCGTCCTCTCTGGGG | CGGTGCAACCACCTTTATCT |
